# Effects of tumor necrosis factor-alpha inhibitors on lipid profiles in patients with psoriasis: a systematic review and meta-analysis

**DOI:** 10.3389/fimmu.2024.1354593

**Published:** 2024-03-04

**Authors:** Liang Su, Chunyan Xu, Hong Huang, Peilian Zhang, Jinrong Wang, Xiaoyong Ouyang, Xuesong Yang, Jianzhou Ye

**Affiliations:** ^1^ Department of Dermatology, The First Affiliated Hospital of Yunnan University of Chinese Medicine, Kunming, China; ^2^ Department of Dermatology, Yunnan Provincial Hospital of Traditional Chinese Medicine, Kunming, China; ^3^ Department of Dermatology, Dongzhimen Hospital, Beijing University of Chinese Medicine, Beijing, China

**Keywords:** tumor necrosis factor alpha (TNF-alpha), tumor necrosis factor alpha inhibitors, psoriasis, lipid profiles, systematic review, meta-analysis

## Abstract

**Background:**

There is no consensus on the effect of tumor necrosis factor-alpha (TNF-alpha) inhibitors on lipid profiles in patients with psoriasis. This study aimed to investigate the effects of TNF-alpha inhibitors on lipid profiles (triglycerides, total cholesterol, low-density lipoprotein, or high-density lipoprotein) in patients with psoriasis.

**Methods:**

We searched PubMed, Embase, and Cochrane Library databases for articles published before October 17, 2023. Four TNF-alpha inhibitors (infliximab, etanercept, adalimumab, and certolizumab) were included in our study. (PROSPERO ID: CRD42023469703).

**Results:**

A total of twenty trials were included. Overall results revealed that TNF-alpha inhibitors elevated high-density lipoprotein levels in patients with psoriasis (WMD = 2.31; 95% CI: 0.96, 3.67; *P* = 0.001), which was supported by the results of sensitivity analyses excluding the effect of lipid-lowering drugs. Subgroup analyses indicated that high-density lipoprotein levels were significantly increased in the less than or equal to 3 months group (WMD = 2.88; 95% CI: 1.37, 4.4; *P* < 0.001), the etanercept group (WMD = 3.4; 95% CI = 1.71, 5.09, *P* < 0.001), and the psoriasis group (WMD = 2.52; 95% CI = 0.57, 4.48, *P* = 0.011). Triglyceride levels were significantly increased in the 3 to 6-month group (WMD = 4.98; 95% CI = 1.97, 7.99, *P* = 0.001) and significantly decreased in the 6-month and older group (WMD = -19.84; 95% CI = -23.97, -15.7, *P* < 0.001). Additionally, Triglyceride levels were significantly increased in the psoriasis group (WMD = 5.22; 95% CI = 2.23, 8.21, *P* = 0.001).

**Conclusion:**

Our results revealed that TNF-alpha inhibitors might temporarily increase high-density lipoprotein levels in patients with psoriasis. However, changes in triglycerides were not consistent among the different durations of treatment, with significant increases after 3 to 6 months of treatment. Future prospective trials with long-term follow-up contribute to confirming and extending our findings.

**Systematic Review Registration:**

https://www.crd.york.ac.uk/PROSPERO/, identifier CRD42023469703.

## 1 Introduction

Psoriasis is a common immune-mediated disease that is mainly associated with the skin and affects approximately 125 million people worldwide (1, 2). Psoriasis is characterized by the formation of silvery-white scaly plaques, and its adverse effect on emotionally and physically relevant quality of life is comparable to other major diseases (1, 3). Of note, psoriasis is a typical inflammatory skin disease whose pathogenesis usually involves the activation of inflammatory processes that have the potential to influence systemic organ responses and functions, which in turn results in the dysfunction of various organs (4–6). Indeed, increasing clinical observations have converged to evidence the high prevalence of co-morbidities in patients with psoriasis (7, 8). Hence, comorbidities in patients with psoriasis often influence the selection of treatment, and it is necessary to consider that certain treatments may ameliorate or exacerbate psoriasis comorbidities (9).

Over the past few decades, lipid disturbances in psoriasis have attracted attention (10). Our previous published meta-analysis also revealed that psoriasis was associated with abnormal apolipoprotein A1 and B levels compared with healthy controls (11). The introduction of biologics has greatly expanded the treatment options for patients with moderate to severe psoriasis (12). Among these biologics, tumor necrosis factor-alpha (TNF-alpha) inhibitors, the first class of approved biologics, have been used for over a decade and have dramatically enhanced clinical outcomes for patients with moderate to severe psoriasis (13, 14). TNF-alpha is a multifunctional cytokine with a series of biological actions that have been reported to regulate and interfere with lipid homeostasis (15). Meanwhile, TNF-alpha inhibitors have been reported to possibly affect lipid metabolism (15). However, there is no consensus on the effect of TNF-alpha inhibitors on lipid profiles in patients with psoriasis (16–18).

Therefore, this study aimed to investigate the effects of TNF-alpha inhibitors on lipid profiles in patients with psoriasis using a systematic review a systematic review and meta-analysis.

## 2 Methods

Our results were reported in accordance with the Preferred Reporting Items for Systematic Reviews and Meta-Analysis (PRISMA) statement (19) (PROSPERO ID: CRD42023469703).

### 2.1 Search strategy

According to a related Cochrane meta-analysis, four TNF-alpha inhibitors (infliximab, etanercept, adalimumab, and certolizumab) were searched (20). We searched PubMed, Embase, and Cochrane Library databases for articles published before October 17, 2023. English language restriction was applied (**Supplementary Appendix 1**). The eligibility of studies was evaluated independently by LS and C-y X, and disagreements were resolved through consultation with J-z Y.

### 2.2 Inclusion and exclusion criteria

The included studies must fulfill the following criteria: 1) patients clinically diagnosed with psoriasis (21); 2) interventions for TNF-alpha inhibitors (infliximab, etanercept, adalimumab, or certolizumab); and 3) outcomes including triglycerides, total cholesterol, low-density lipoprotein, or high-density lipoprotein. Furthermore, the exclusion criteria were as follows: 1) reporting study populations include psoriasis combined with other autoimmune diseases; 2) significant changes in medications for systemic treatment of psoriasis during the observation period compared to pre-treatment; and 2) letters, editorials, books, or studies that did not provide sufficient data.

### 2.3 Data extraction and quality assessment

We extracted the following data from each included study: first author, country, study design, publication year, type of psoriasis, sample size, age, psoriasis area and severity index (PASI), interventions (the type of TNF-alpha inhibitors), duration of treatment, and lipid profiles (triglycerides, total cholesterol, low-density lipoprotein, or high-density lipoprotein) data. The methodological index for non-randomized studies (MINORS) was employed to assess the quality of included studies, which consisted of eight items (22). Each item was assigned a score ranging from 0 to 2, with high scores representing adequate reporting. Two reviewers (LS and C-y X) independently extracted the data and assessed the risk of bias (RoB), with any disagreements resolved by a third reviewer (J-z Y).

### 2.4 Statistical analysis

Considering that random-effects model provides more conservative results, we performed a meta-analysis with a random-effects model using Stata15 software (23, 24). To evaluate the effects of TNF-alpha inhibitors on lipid profiles, the results were presented as weighted mean differences (WMDs) with their 95% confidence intervals (CIs). In the overall results, when studies reported results for different intervention durations, our analyses utilized data for the longest intervention duration. When patients with psoriasis were divided into subgroups according to the type of TNF-alpha inhibitors, we calculated the pooled mean and standard deviation (SD), as suggested by the Cochrane Handbook (25). Study heterogeneity was assessed using the Cochran’s Q and *I* ^2^ statistics (26). Publication bias was assessed using the funnel plot and Egger’s test (27). We conducted sensitivity analyses by removing each study in turn. Additional sensitivity analyses were performed only included in studies that reported exclusion of lipid-lowering drugs or no significant change in lipid-lowering drugs during the observation period. Subgroup analyses were performed according to the type of TNF-alpha inhibitor and duration of intervention. Additional analyses were performed for psoriasis type (psoriasis or psoriatic arthritis) and PASI scores for psoriasis [mean or median PASI more than 10, which represents moderate to severe psoriasis (28)]. Statistical significance was defined as *P* < 0.05.

## 3 Results

### 3.1 Search results

**Figure 1:**
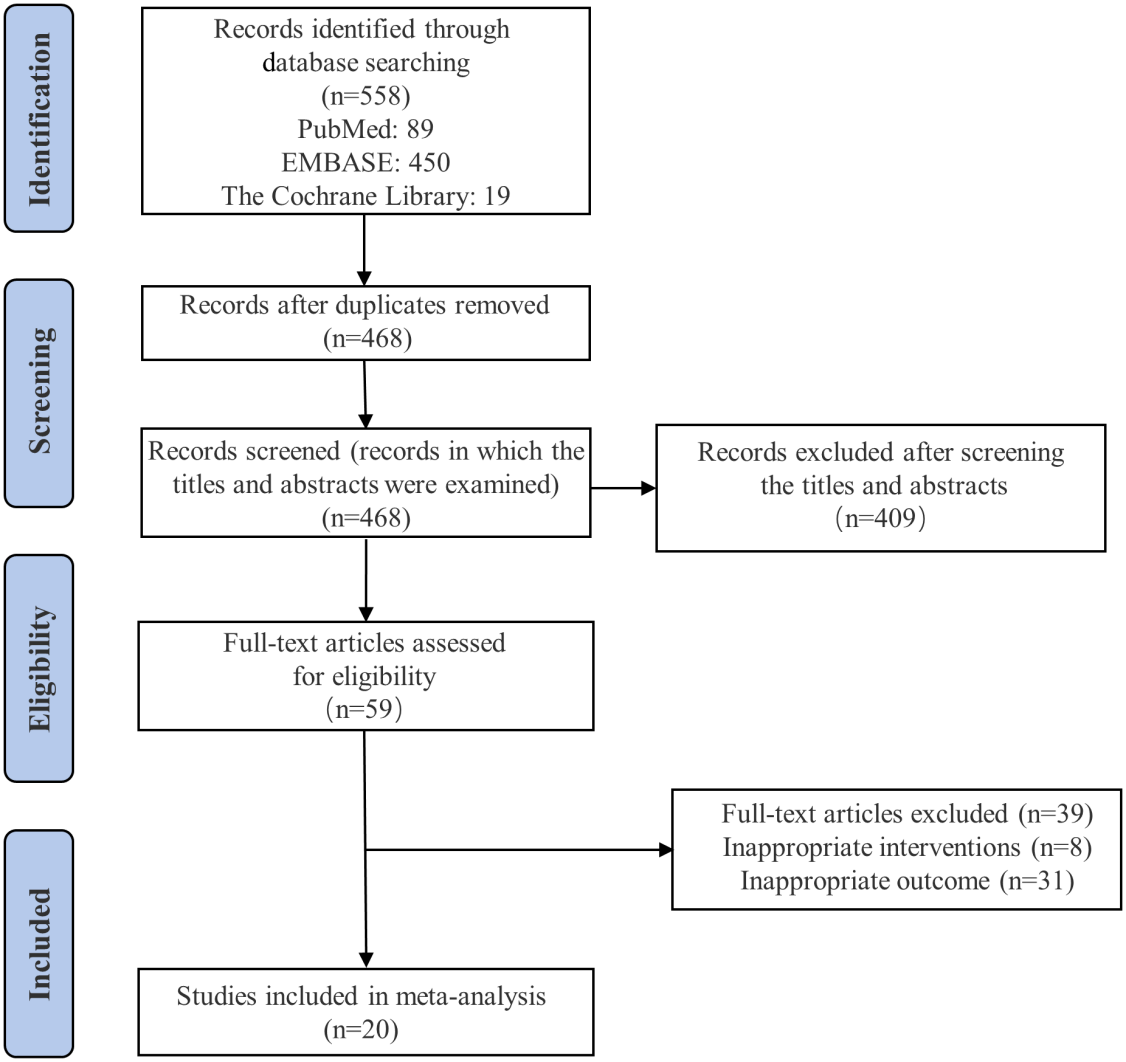
Flow diagram for the study selection process.

The literature search identified 558 publications, of which 59 were fully reviewed and 20 studies (29–48) were finally eligible for inclusion. **Figure 1** and **Supplementary Appendix 2** illustrate the detailed information of the literature selection procedure.

### 3.2 Study characteristics and quality

The baseline characteristics of the included studies are shown in **Table 1**. The 20 studies included were conducted in 11 countries. The mean (median) PASI of the participants was 6-21.2, and the mean age of the participants was 34-56 years. The duration of the intervention ranged from 2 to 36 months. Sixteen studies reported data on triglycerides. Fifteen studies reported data on total cholesterol and high-density lipoprotein respectively. Twelve studies reported data on low-density lipoprotein. Fifteen studies illustrated specific types of TNF-alpha inhibitors. Nine studies reported exclusion of lipid-lowering drugs or no significant change in lipid-lowering drugs during the observation period. The RoB assessment by MINORS is shown in **Table 1**.

**Table 1:** Baseline characteristics of the included studies.

Study	Study design	Location	Sample size (n)	F/M	Age	Pso type	PASI	Treatment	Outcome	Quality
Bacchetti et al., 2013 (29)	Nonrandomized	Italy	23	13/10	47.5	Pso	19.4	Etanercept for 24 weeks	Trig, TC, HDL, LDL	12
Campanati et al., 2017 (30)	Nonrandomized	Italy	20	9/11	56	Pso	17.95	Adalimumab or Etanercept for 24 weeks	Trig, TC, HDL	11
Castro et al., 2011 (31)	Prospective	Brazil	15	7/8	41.9	PsA	NA	Infliximab for 3 months	Trig, TC, HDL, LDL	10
Demir et al., 2020 (32)	Prospective	Turkey	14	2/12	34	Pso and PsA	15.38	Adalimumab and etanercept for 12 weeks	Trig, HDL, LDL	9
Ehsani et al., 2016 (33)	Prospective	Iran	25	7/18	36.91	Pso	NA	Infliximab for 24 weeks	Trig, TC, HDL, LDL	13
Elnabawi et al., 2019 (34)	Prospective	USA	48	17/31	49.8	Pso	8	Adalimumab or etanercept for 12 months	TC, HDL, LDL	13
Gisondi et al., 2013 (35)	Retrospective	Italy	2855	NA	NA	Pso	NA	Adalimumab or Etanercept or Infliximab for 16 weeks	Trig, TC, LDL	11
Gisondi et al., 2013 (36)	Prospective	Italy	40	17/23	49.9	Pso and PsA	14.1	Infliximab for 12 months	Trig	11
Hagino et al., 2023 (37)	Retrospective	Japan	56	12/44	54	Pso and PsA	9.05	Adalimumab or infliximab certolizumab pegol for 52 weeks	TC, HDL, LDL	11
Holzer et al., 2021 (38)	Randomized	Austria	33	5/28	46.3	Pso	16.3	Adalimumab for 6 months	Trig, TC, HDL, LDL	12
Mehta et al., 2018 (39)	Randomized	USA	33	9/24	44.15	Pso and PsA	17.4	Adalimumab for 52 weeks	TC	13
Merlo et al., 2020 (40)	Prospective	Italy	31	10/21	55	Pso	17.2	Adalimumab or etanercept for 12 months	Trig, HDL	9
Olejniczak-Staruch et al. 2021 (41)	Nonrandomized	Poland	37	17/20	49.7	Pso and PsA	20.1	Adalimumab or Etanercept or Infliximab for 36 months	Trig, TC, HDL, LDL	10
Puig et al., 2014 (42)	Prospective	Spain	273	83/190	43.9	Pso and PsA	21.2	Etanercept for 12 weeks	Trig, TC, HDL, LDL	10
Ramonda et al., 2014 (43)	Prospective	Italy	32	15/17	51	PsA	NA	Adalimumab or Etanercept or Infliximab for 24 months	Trig, TC, HDL, LDL	11
Skroza et al., 2013 (44)	Nonrandomized	Italy	20	11/9	47.85	Pso	6	Etanercept for 3 months	Trig, HDL	8
Spanakis et al., 2006 (45)	Prospective	Greece	10	6/4	42.5	PsA	NA	Infliximab for 6 months	TC, HDL	10
Takamura et al., 2018 (46)	Retrospective	Japan	18	17/1	41.4	Pso	16	Infliximab for 7 months	Trig, TC	10
Wu et al., 2014 (47)	Retrospective	USA	1274	618/656	46.7	Pso and PsA	NA	Etanercept or adalimumab or infliximab for 12 months	Trig, HDL, LDL	11
Zhao et al., 2022 (48)	Prospective	China	13	2/11	38.69	Pso	20.54	Adalimumab for 3 months	Trig, TC	9

F/M, Female/Male; HDL, High-density lipoprotein; LDL, Low-density lipoprotein; NA, Not applicable; PsA, Psoriatic arthritis; Pso, Psoriasis; PASI, Psoriasis area and severity index; TC, Total cholesterol; Trig, Triglycerides.

### 3.3 Effects of TNF-alpha inhibitors on triglycerides, total cholesterol, low-density lipoprotein, and high-density lipoprotein in patients with psoriasis

**Figure 2:**
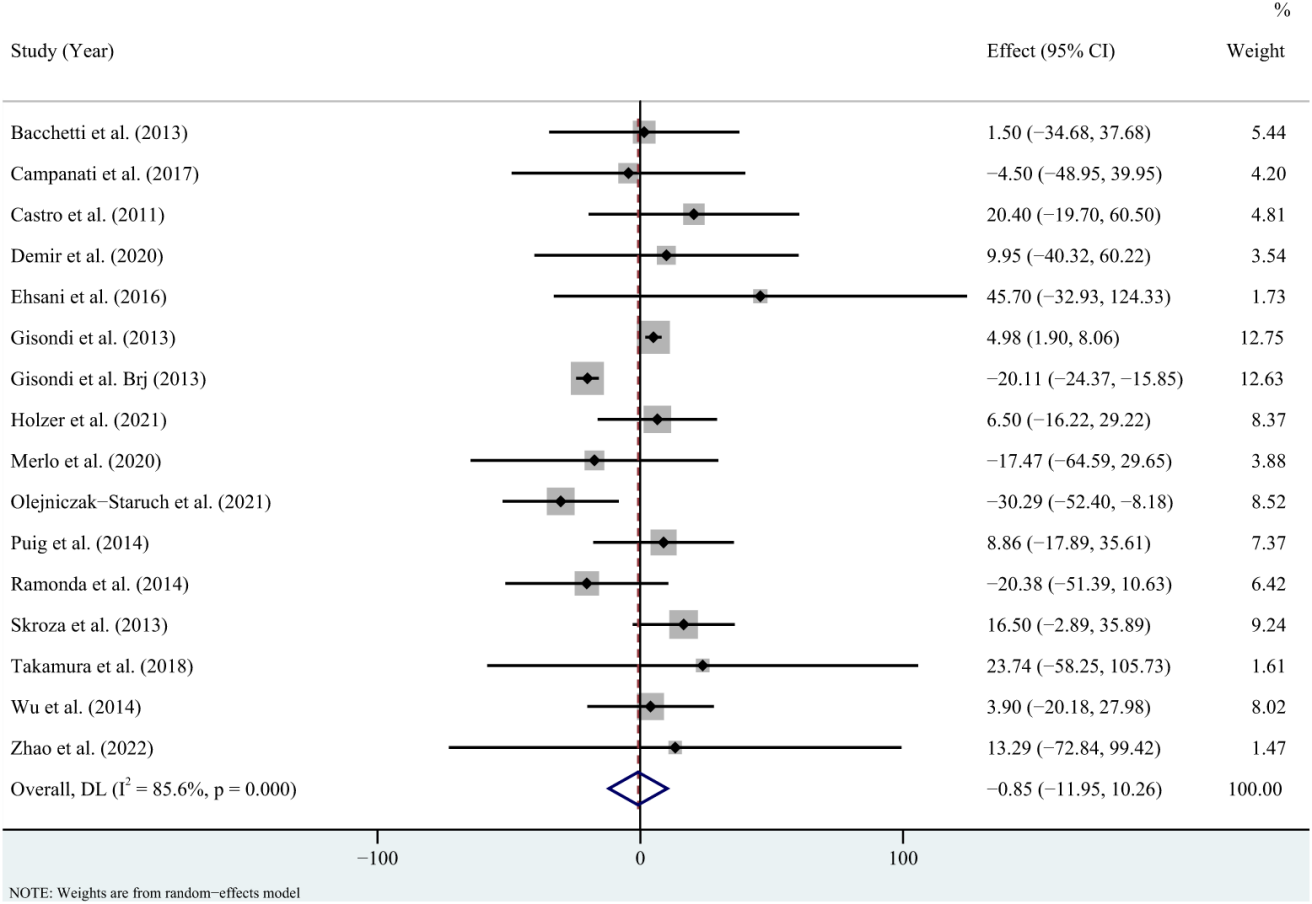
Forest plot for the effect of TNF-alpha inhibitors on triglycerides in patients with psoriasis (weighted mean difference).

**Figure 3:**
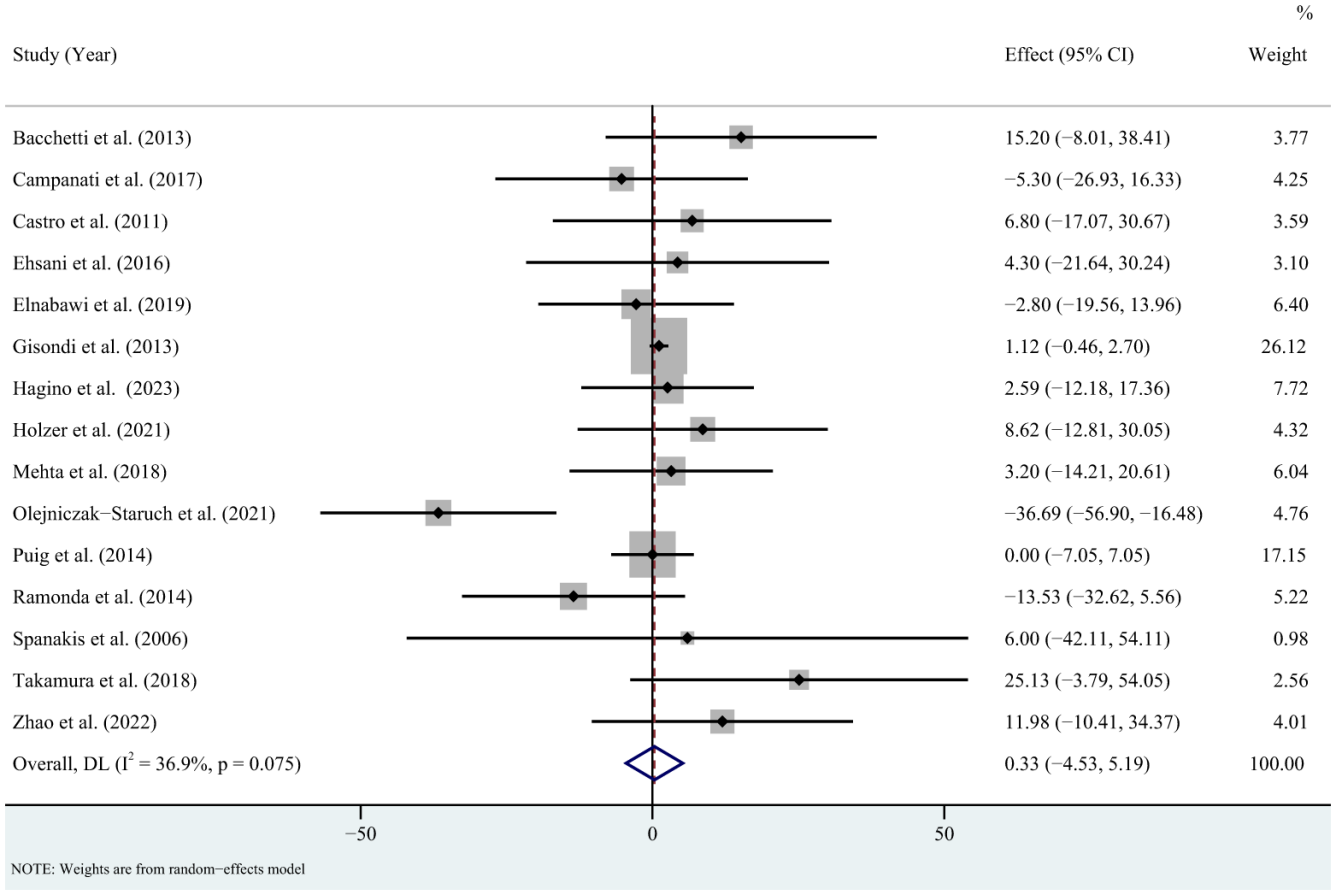
Forest plot for the effect of TNF-alpha inhibitors on total cholesterol in patients with psoriasis (weighted mean difference).

**Figure 4:**
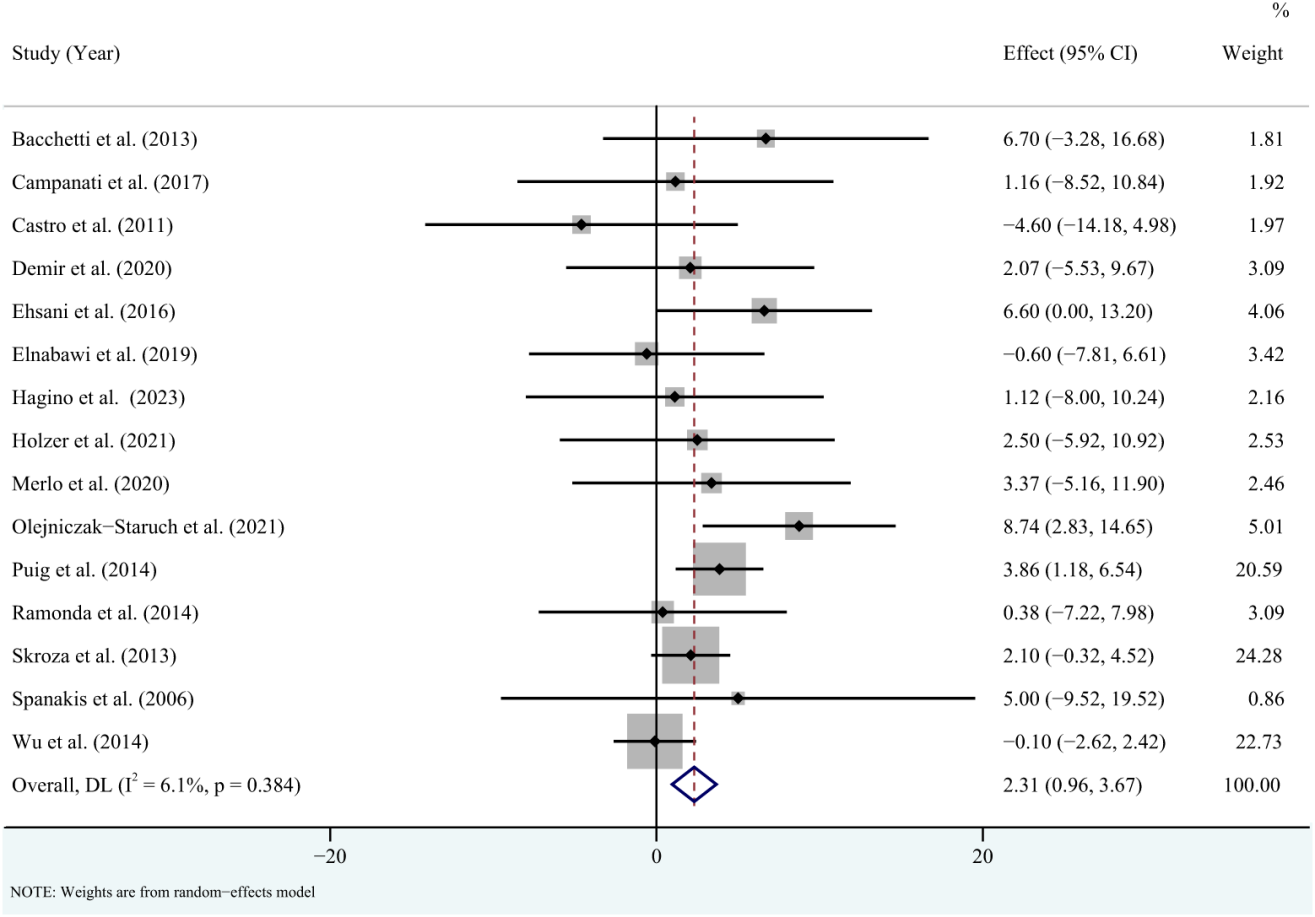
Forest plot for the effect of TNF-alpha inhibitors on high-density lipoprotein in patients with psoriasis (weighted mean difference).

**Figure 5:**
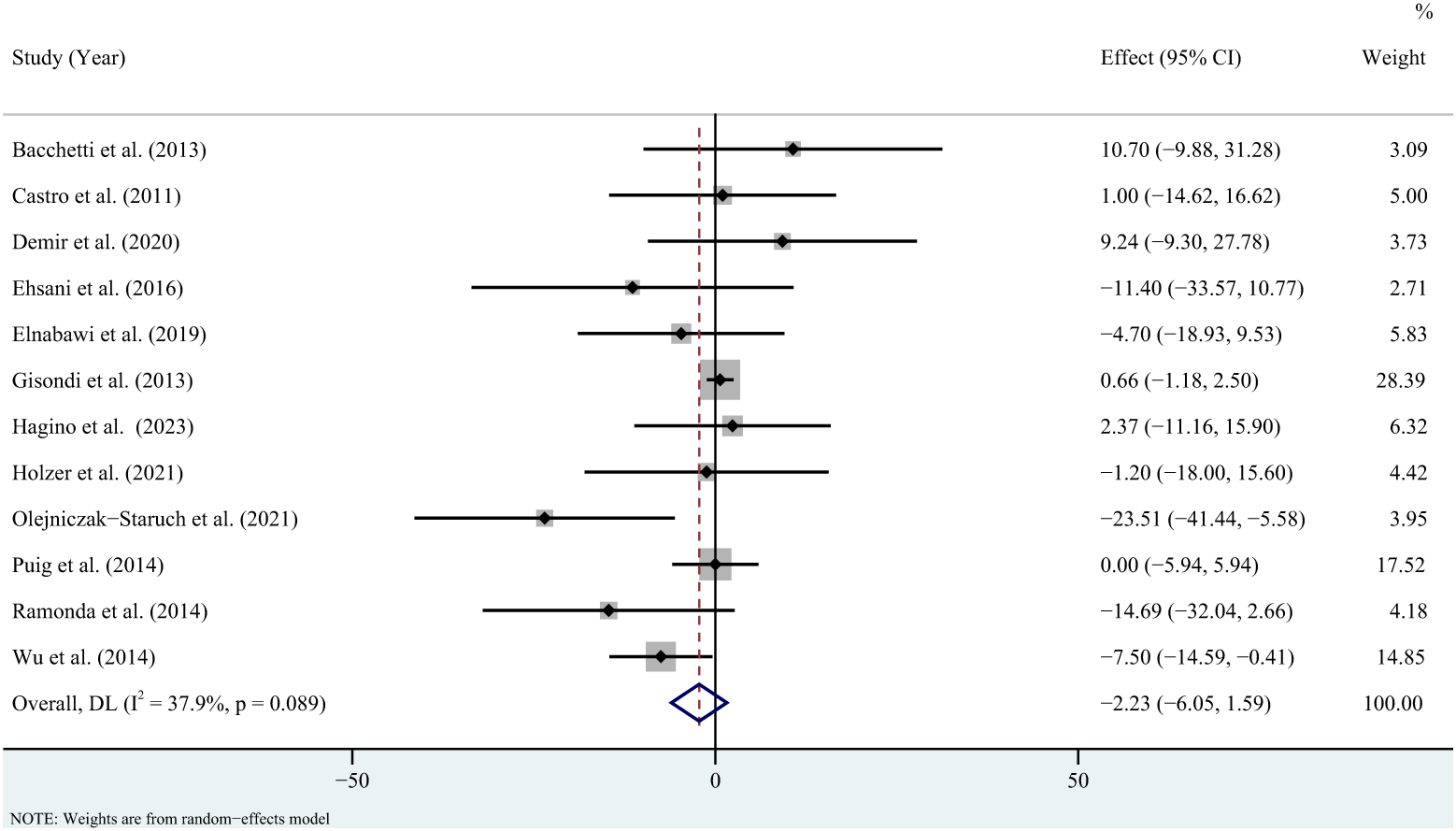
Forest plot for the effect of TNF-alpha inhibitors on low-density lipoprotein in patients with psoriasis (weighted mean difference).

For triglycerides, the meta-analysis showed that pooled WMD was -0.85 (95% CI: -11.95, 10.26, *P* = 0.881; *I* ^2 = ^85.6%, *P* < 0.001) (**Figure 2**). Sensitivity analysis revealed that this result did not change significantly when any individual study was removed (**Supplementary Figure 1**). For total cholesterol, the meta-analysis showed that pooled WMD was 0.33 (95% CI: -4.53, 5.19, *P* = 0.893; *I* ^2 = ^36.9%, *P* = 0.075) (**Figure 3**). Sensitivity analysis revealed that this result did not change significantly when any individual study was removed (**Supplementary Figure 2**). For high-density lipoprotein, the meta-analysis showed that pooled WMD was 2.31 (95% CI: 0.96, 3.67, *P* = 0.001; *I* ^2 = ^6.1%, *P* = 0.384) (**Figure 4**). Sensitivity analysis revealed that this result did not change significantly when any individual study was removed (**Supplementary Figure 3**). For low-density lipoprotein, the meta-analysis showed that pooled WMD was -2.23 (95% CI: -6.05, 1.59, *P* = 0.253; *I* ^2 = ^37.9%, *P* = 0.089) (**Figure 5**). Sensitivity analysis revealed that this result did not change significantly when any individual study was removed (**Supplementary Figure 4**). We conducted additional sensitivity analyses, including only studies that reported exclusion of lipid-lowering drugs or no significant change in lipid-lowering drugs during the observation period. The findings were consistent with the overall results (**Supplementary Figure 5**-**8**).

### 3.4 Subgroup analysis according to duration of intervention

The subgroup analysis revealed that triglycerides levels were not significantly increased in the less than or equal to 3 months group (WMD = 11.82; 95% CI = -2.8, 26.44, *P* = 0.113; *I* ^2 = ^90.9%), while were significantly increased in the 3 to 6 months group (WMD = 4.98; 95% CI = 1.97, 7.99, *P* = 0.001; *I* ^2 = ^0%) and decreased in the greater than 6 months group (WMD = -19.84; 95% CI = -23.97, -15.7, *P* < 0.001; *I* ^2 = ^0%) (**Supplementary Figure 9**). Total cholesterol levels did not change significantly in the less than or equal to 3 months group (WMD = 1.67; 95% CI = -3.1, 6.45, *P* = 0.492; *I* ^2 = ^30.3%), the 3 to 6 months group (WMD = 1.21; 95% CI = -0.36, 2.77, *P* = 0.13; *I* ^2 = ^0%) and the greater than 6 months group (WMD = -1.77; 95% CI = -11.08, 7.55, *P* = 0.71; *I* ^2 = ^31.9%) (**Supplementary Figure 10**). High-density lipoprotein levels were significantly increased in the less than or equal to 3 months group (WMD = 2.88; 95% CI = 1.37, 4.4, *P* < 0.001; *I* ^2 = ^0%), while were not significantly increased in the 3 to 6 months group (WMD = 1.39; 95% CI = -0.87, 3.65, *P* = 0.229; *I* ^2 = ^2.2%) and the greater than 6 months group (WMD = 2.16; 95% CI = -1.01, 5.33, *P* = 0.182; *I* ^2 = ^0%) (**Supplementary Figure 11**). Low-density lipoprotein levels did not change significantly in the less than or equal to 3 months group (WMD = 1.12; 95% CI = -3.25, 5.49, *P* = 0.617; *I* ^2 = ^36.2%), the 3 to 6 months group (WMD = -1.85; 95% CI = -7.3, 3.61, *P* = 0.507; *I* ^2 = ^41.6%) and the greater than 6 months group (WMD = -5.19; 95% CI = -13.04, 2.66, *P* = 0.195; *I* ^2 = ^0%) (**Supplementary Figure 12**).

### 3.5 Subgroup analysis according to type of TNF-alpha inhibitor

The subgroup analysis revealed that triglycerides levels did not change significantly in the adalimumab group (WMD = -5.31; 95% CI = -15.47, 4.85, *P* = 0.305; *I* ^2 = ^0%), the etanercept group (WMD = 1.67; 95% CI = -12.51, 15.85, *P* = 0.817; *I* ^2 = ^55.6%) and the infliximab group (WMD = -7.45; 95% CI = -21.64, 6.74, *P* = 0.303; *I* ^2 = ^79.4%) (**Supplementary Figure 13**). Total cholesterol levels did not change significantly in the adalimumab group (WMD = 7.87; 95% CI = -0.91, 16.65, *P* = 0.079; *I* ^2 = ^28.6%), the etanercept group (WMD = -0.46; 95% CI = -9.71, 8.79, *P* = 0.923; *I* ^2 = ^72.1%) and the infliximab group (WMD = -1.57; 95% CI = -11.91, 8.76, *P* = 0.765; *I* ^2 = ^38.6%) (**Supplementary Figure 14**). High-density lipoprotein levels were significantly increased in the etanercept group (WMD = 3.4; 95% CI = 1.71, 5.09, *P* < 0.001; *I* ^2 = ^0%), while were not significantly increased in the adalimumab group (WMD = 2.85; 95% CI = -2.8, 8.5, *P* = 0.323; *I* ^2 = ^0%) and the infliximab group (WMD = 6.2; 95% CI = -3.27, 15.67, *P* = 0.199; *I* ^2 = ^84.4%) (**Supplementary Figure 15**). Low-density lipoprotein levels did not change significantly adalimumab group (WMD = 2.67; 95% CI = -7.63, 12.98, *P* = 0.611; *I* ^2 = ^27.1%), the etanercept group (WMD = -0.01; 95% CI = -5.67, 5.65, *P* = 0.998; *I* ^2 = ^50.8%) and the infliximab group (WMD = -9.16; 95% CI = -21.18, 2.86, *P* = 0.135; *I* ^2 = ^75.6%) (**Supplementary Figure 16**).

### 3.6 Additional analyses

According to the PASI scores for psoriasis (mean or median PASI more than 10), the findings were consistent with the overall results (**Supplementary Figures 17**-**20**). According to the psoriasis type (psoriasis or psoriatic arthritis), the results of total cholesterol and low-density lipoprotein were consistent with the overall results (**Supplementary Figures 21**, **22**). Triglycerides levels were significantly increased in the psoriasis group (WMD = 5.22; 95% CI = 2.23, 8.21, *P* = 0.001; *I* ^2 = ^0%), while were not change significantly in the psoriatic arthritis group (WMD = -2.05; 95% CI = -41.81, 37.7, *P* = 0.919; *I* ^2 = ^59.8%) (**Supplementary Figure 23**). High-density lipoprotein levels were significantly increased in the psoriasis group (WMD = 2.52; 95% CI = 0.57, 4.48, *P* = 0.011; *I* ^2 = ^0%), while were not change significantly in the psoriatic arthritis group (WMD = -0.6; 95% CI = -6.11, 4.9, *P* = 0.83; *I* ^2 = ^0%) (**Supplementary Figure 24**).

### 3.7 Publication bias

For the overall results, a funnel plot showed that a possible publication bias may exist in triglycerides (**Supplementary Figure 25**) and total cholesterol (**Supplementary Figure 26**), although Egger’s test was not statistically significant in triglycerides (*P* = 0.835; **Supplementary Figure 27**) and total cholesterol (*P* = 0.931; **Supplementary Figure 28**). There was no obvious funnel plot asymmetry for the high-density lipoprotein (**Supplementary Figure 29**), and low-density lipoprotein (**Supplementary Figure 30**). Furthermore, Egger’s test revealed no statistical evidence of publication bias in high-density lipoprotein (*P* = 0.641; **Supplementary Figure 31**) and low-density lipoprotein (*P* = 0.188; **Supplementary Figure 32**).

## 4 Discussion

In this study, we evaluated the effect of TNF-alpha inhibitors on lipid profiles (triglycerides, total cholesterol, low-density lipoprotein, and high-density lipoprotein) in patients with psoriasis. The overall findings revealed that TNF-alpha inhibitors elevated high-density lipoprotein levels in patients with psoriasis, which was supported by the results of sensitivity analyses excluding the effect of lipid-lowering drugs. We also performed subgroup analyses according to the type of TNF-alpha inhibitor, treatment duration, PASI scores, and psoriasis type. High-density lipoprotein levels were significantly increased in the less than or equal to 3 months group, the etanercept group, and the psoriasis group. Changes in triglyceride levels were not consistent among the different durations of treatment. Specifically, triglycerides were significantly increased in the 3 to 6-month group and significantly decreased in the 6-month and older group. In addition, triglycerides significantly increased the psoriasis group.

The negative relationship between high-density lipoprotein levels and the risk of coronary heart disease dates back to the 1950s, and it remains an important and powerful risk marker for the developing risk of atherosclerotic cardiovascular disease (49). The increase in high-density lipoprotein levels may represent a cardioprotective effect (50). However, a previous meta-analysis found no significant difference in the rate of major adverse cardiovascular events in psoriasis patients treated with TNF-alpha inhibitors compared with placebo (51). Thus, the increase of high-density lipoprotein levels observed in this meta-analysis may limited. Actually, our subgroups also revealed that high-density lipoprotein levels were significantly elevated only in the less-than-or-equal-to-3-months group. On the other hand, high-density lipoprotein levels were significantly increased in the etanercept group, while were not significantly increased in the adalimumab group and the infliximab group. Head-to-head trials comparing the effectiveness of etanercept, adalimumab, and infliximab in the treatment of psoriasis are limited, and there is a lack of consensus on the difference in effectiveness between them (52–54). Thus, the elevated high-density lipoprotein levels in the etanercept group may be explained by the fact that this result was primarily driven by the studies from Puig, L (42). and Skroza, N (44)., both of which were treated for three months. Conversely, low-density lipoprotein is the major atherogenic lipoprotein and has been reported not to modify in patients with rheumatoid arthritis after treatment with TNF-alpha inhibitors (50). Similarly, the decrease of low-density lipoprotein levels observed in this meta-analysis lacked statistical significance. Total cholesterol consists mainly of low-density lipoprotein and high-density lipoprotein cholesterol (55, 56). Hence, a potential explanation for the total cholesterol results observed in this meta-analysis is the limited increase in high-density lipoprotein levels and the lack of statistical significance of the decrease in low-density lipoprotein levels. TNF-alpha inhibitors have been reported to exhibit a tendency to increase triglycerides in the treatment of patients with rheumatoid arthritis (57). A recent meta-analysis investigating the effect of TNF-alpha inhibitors on the lipid profile of patients with rheumatic diseases showed that changes in triglycerides were not consistent among the different time point assessments (58). Specifically, triglycerides were marginally significantly increased at short-term and middle-term assessments and significantly increased at the long-term assessment (58). Our subgroup analysis revealed that triglyceride levels were not significantly increased in the less than or equal to 3 months group, while were significantly increased in the 3 to 6 months group and decreased in the greater than 6 months group. Considering the large change in effect size in the greater than 6 months group, a potential explanation for the triglycerides results may be related to the use of lipid-lowering drugs. Actually, more than half of the studies we included did not report data on the use of lipid-lowering drugs. The bias in the efficacy of lipid-lowering drugs may provide a plausible explanation for the significant increase in the 3 to 6-month group and the significant decrease in the greater than 6-month group. As for the differences in results between psoriasis and psoriatic arthritis, one potential reason may be that their pathophysiological mechanisms are not entirely identical (59, 60). In addition, psoriatic arthritis may be comorbid with more severe complications and respond relatively inadequately to biologics, which may be another potential explanation for our results (61). Notably, this result needs further investigation, especially given the small number of trials included in the psoriatic arthritis group.

Psoriasis is a typical inflammatory skin disease (62). An underlying mechanism for our results may be related to inflammation and lipid metabolism, which has been reported in other inflammatory skin diseases (63, 64). Hidradenitis suppurativa is a chronic inflammatory skin disease (63). Excessive obesity, especially in visceral depots, is connected with adipose tissue dysfunction, which manifests as a potentially pro-inflammatory state (63). The development of hidradenitis suppurativa may be partly driven by excess visceral adiposity and chronic inflammation (63). Therefore, the assessment of metabolic risk may be an important component in the clinical management of inflammatory skin diseases (64). High-density lipoprotein possesses anti-inflammatory effects, while inflammation also reduces high-density lipoprotein levels (65). Correspondingly, psoriasis patients were noted to have reduced high-density lipoprotein levels than healthy controls (65). It has been reported that cytokine expression can reduce high-density lipoprotein levels during inflammation, and the specific mechanism may be related to mediating the downregulation of peroxisome proliferator-activated receptor gamma expression (57, 66). Actually, TNF-alpha is a key inflammatory mediator, and it has been reported to interfere with cholesterol metabolism possibly (67, 68). TNF-alpha inhibitors are known to possess anti-inflammatory effects. Thus, the increase of high-density lipoprotein levels observed in this meta-analysis may be associated with TNF-alpha inhibitors alleviating inflammation in patients with psoriasis. In addition, TNF-alpha is involved in body weight homeostasis by enhancing lipolysis and depressing adipogenesis, and TNF-alpha inhibitor therapy appears to be linked to increased body weight in psoriasis patients (67). Further studies have shown that TNF-alpha increases lipolysis and promotes muscle cell catabolism by mediating the activation of the nuclear transcription factor NF-kB (30). In contrast, TNF-alpha inhibitors possess the ability to induce muscle and adipocytes to take up glucose and convert it to triglycerides and glycogen (58). In rheumatoid arthritis or ankylosing spondylitis patients, long-term TNF-alpha inhibitors have also been reported to be associated with a significant increase in fat mass, with a shift to the visceral region (69). Notably, classical methotrexate therapy for psoriasis did not appear to significantly increase body mass index (47). Obesity, in particular abdominal obesity, seems to determine triglyceride levels (70, 71). Therefore, the total cholesterol results observed in this meta-analysis may be related to the effect of TNF-alpha inhibitors on the metabolism of adipose and muscle tissue.

To the best of our knowledge, this study is the first meta-analysis to evaluate the effect of TNF-alpha inhibitors on lipid profiles (triglycerides, total cholesterol, low-density lipoprotein, and high-density lipoprotein) in patients with psoriasis. Meanwhile, the heterogeneity of most results is acceptable. Certainly, this study has limitations that cannot be denied. Firstly, we must acknowledge that this study lacked a placebo control group. Therefore, the influence of study design cannot be excluded. However, we cannot deny the fact that the ethics of using placebo control in patients with moderate to severe psoriasis are widely debated (72). Thus, placebo-controlled data are limited. A patient registry is a structured set of observational data on a population defined by a particular disease or condition, which contains relatively broader inclusion and exclusion criteria than randomized clinical trials (73). Hence, patient registries have larger sample sizes, which may increase the generalizability of results to clinical practice (73). Actually, patient registries have gained attention in psoriasis research (74, 75). Establishing a specific patient registry of biologics for the treatment of psoriasis may contribute to extending our results. Second, owing to limited data, our study failed to investigate the effect of certolizumab in subgroup analyses. Third, although we performed subgroup analyses according to the type of TNF-alpha inhibitors, we did not compare the effects of different TNF-alpha inhibitors. Thus, network meta-analysis contributes to extending our findings. Finally, the prevalence of psoriasis varies significantly in the global population, suggesting that there may be population differences in the pathogenesis of psoriasis (76). Hence, whether our findings vary across regions and countries is a question worth exploring. However, limited data restricted our ability to perform stratified analyses according to region and nation. Further well-designed controlled studies contribute to confirming and extending our findings.

## 5 Conclusions

Our results revealed that TNF-alpha inhibitors might temporarily increase high-density lipoprotein levels in patients with psoriasis. However, changes in triglycerides were not consistent among the different durations of treatment, with significant increases after 3 to 6 months of treatment. Our findings emphasized the importance of screening lipids in the treatment of psoriasis with TNF-alpha inhibitors. Considering the limitations of our study, future prospective trials with long-term follow-up contribute to confirming and extending our findings.

## Data availability statement

The original contributions presented in the study are included in the article/**Supplementary Materials**, further inquiries can be directed to the corresponding author/s.

## Author contributions

LS: Data curation, Formal analysis, Writing – original draft. CX: Data curation, Formal analysis, Writing – review and editing. HH: Writing – review and editing. PZ: Writing – review and editing. JW: Writing – review and editing. XO: Conceptualization, Methodology, Project administration, Writing – review and editing. XY: Conceptualization, Methodology, Project administration, Writing – review and editing. JY: Conceptualization, Methodology, Project administration, Writing – review and editing.
