# Supplementary material for: Effects of tumor necrosis factor-alpha inhibitors on lipid profiles in patients with psoriasis: a systematic review and meta-analysis
Source: Front Immunol. 2024 Mar 4;15:1354593. doi: 10.3389/fimmu.2024.1354593 (PMC10944886; doi:10.3389/fimmu.2024.1354593)
Supplement: Supplementary file 1 [file DataSheet_1.docx]

**Effects of tumor necrosis factor alpha inhibitors on lipid profiles in patients with psoriasis: A systematic review and meta-analysis**

Liang Su et al.

**“Online Supplementary Material”**

Supplemental Appendix 1:

Full search strategy (17th October 2023): PubMed

| **Searches** |
| --- |
| #1 "Psoriasis"[MeSH Terms] OR "Psoriasis"[Title/Abstract] OR "Psoriases"[Title/Abstract]  #2 "Triglycerides"[MeSH Terms] OR "Cholesterol"[MeSH Terms] OR "lipoproteins, ldl"[MeSH Terms] OR "lipoproteins, hdl"[MeSH Terms] OR "Triglycerides"[Title/Abstract] OR "Triglyceride"[Title/Abstract] OR "Triacylglycerols"[Title/Abstract] OR "Triacylglycerol"[Title/Abstract] OR "Cholesterol"[Title/Abstract] OR "Epicholesterol"[Title/Abstract] OR "low density lipoprotein"[Title/Abstract] OR "high density lipoprotein"[Title/Abstract] OR "Lipoproteins"[Title/Abstract] OR "Lipoprotein"[Title/Abstract]  #3 "Tumor Necrosis Factors"[MeSH Terms] OR "Infliximab"[MeSH Terms] OR "Etanercept"[MeSH Terms] OR "Adalimumab"[MeSH Terms] OR "Certolizumab Pegol"[MeSH Terms] OR "Tumor Necrosis Factors"[Title/Abstract] OR "tumor necrosis factor"[Title/Abstract] OR "anti-TNF"[Title/Abstract] OR "TNF-α"[Title/Abstract] OR "TNF-alpha"[Title/Abstract] OR "Infliximab"[Title/Abstract] OR "Inflectra"[Title/Abstract] OR "Remicade"[Title/Abstract] OR "Renflexis"[Title/Abstract] OR "Etanercept"[Title/Abstract] OR "Erelzi"[Title/Abstract] OR "Enbrel"[Title/Abstract] OR "Adalimumab"[Title/Abstract] OR "Humira"[Title/Abstract] OR "Amjevita"[Title/Abstract] OR "Cyltezo"[Title/Abstract] OR "Certolizumab"[Title/Abstract] OR "Cimzia"[Title/Abstract]  #4 #1 AND #2 AND #3  #5 "animals"[MeSH Terms] NOT "humans"[MeSH Terms]  #6 #4 NOT #5  #7 limit #6 to English language |

Supplemental Appendix 2: Full-text publications excluded with reason

| **Online Supplementary Reference** | **Reason for exclusion** |
| --- | --- |
| (1-8) | Inappropriate interventions |
| (9-39) | Inappropriate outcome |

**Reference**

1. Sattar N, Crompton P, Cherry L, Kane D, Lowe G, McInnes IB. Effects of tumor necrosis factor blockade on cardiovascular risk factors in psoriatic arthritis: a double-blind, placebo-controlled study. Arthritis and rheumatism. 2007;56(3):831-9.

2. Peters MJ, Watt P, Cherry L, Welsh P, Henninger E, Dijkmans BA, et al. Lack of effect of TNFalpha blockade therapy on circulating adiponectin levels in patients with autoimmune disease: results from two independent prospective studies. Annals of the rheumatic diseases. 2010;69(9):1687-90.

3. Melgäo SSC, da Silva Junior GB, Dantas AMM, Vasconcelos AMA, de Siqueira VR, Vieira APF, et al. Evaluation of renal function in patients with psoriasis using immunobiologicals. Anais Brasileiros de Dermatologia. 2013;88(4):667-9.

4. Lestre S, Diamantino F, Veloso L, Fidalgo A, Ferreira A. Effects of etanercept treatment on lipid profile in patients with moderate-to-severe chronic plaque psoriasis: a retrospective cohort study. European journal of dermatology : EJD. 2011;21(6):916-20.

5. Egeberg A, Wu JJ, Korman N, Solomon JA, Goldblum O, Zhao F, et al. Ixekizumab treatment shows a neutral impact on cardiovascular parameters in patients with moderate-to-severe plaque psoriasis: Results from UNCOVER-1, UNCOVER-2, and UNCOVER-3. J Am Acad Dermatol. 2018;79(1):104-9.e8.

6. Dey AK, Joshi AA, Chaturvedi A, Lerman JB, Aberra TM, Rodante JA, et al. Association Between Skin and Aortic Vascular Inflammation in Patients With Psoriasis: A Case-Cohort Study Using Positron Emission Tomography/Computed Tomography. JAMA cardiology. 2017;2(9):1013-8.

7. Costa L, Caso F, Atteno M, Del Puente A, Darda MA, Caso P, et al. Impact of 24-month treatment with etanercept, adalimumab, or methotrexate on metabolic syndrome components in a cohort of 210 psoriatic arthritis patients. Clinical rheumatology. 2014;33(6):833-9.

8. Agca R, Heslinga M, Kneepkens EL, van Dongen C, Nurmohamed MT. The Effects of 5-year Etanercept Therapy on Cardiovascular Risk Factors in Patients with Psoriatic Arthritis. The Journal of rheumatology. 2017;44(9):1362-8.

9. Madani AN, Al-Saif FM, Alzamil LR, Almazroua AM, Alfurayh NA, Aldokhayel SD, et al. Monitoring the effect of TNF-alpha inhibitors on laboratory parameters and adverse effects in different diseases: a retrospective, single-center study. Annals of Saudi Medicine. 2022;42(5):309-18.

10. Zdanowska N, Owczarczyk-Saczonek AB, Czerwińska J, Nowakowski JJ, Kozera-żywczyk A, Owczarek W, et al. The effect of cigarette smoking on serum levels of regulatory cytokines and molecules involved in atherogenesis during systemic treatment of psoriasis – results of a preliminary study. Polish Annals of Medicine. 2021;28(2):141-9.

11. Zdanowska N, Owczarczyk-Saczonek A, Czerwińska J, Nowakowski JJ, Kozera-Żywczyk A, Owczarek W, et al. Methotrexate decreases oxidized low-density lipoprotein serum levels in patients with plaque psoriasis ⇓ results of a preliminary study. Acta Poloniae Pharmaceutica - Drug Research. 2021;78(1):121-7.

12. Zangrilli A, Bavetta M, Scaramella M, Bianchi L. Long-term treatment of psoriatic patients with adalimumab reduces disease severity and maintains a favorable lipid pattern and a low Atherogenic Index. Giornale italiano di dermatologia e venereologia : organo ufficiale, Societa italiana di dermatologia e sifilografia. 2018;153(2):146-54.

13. Vandikas MS, Landin-Wilhelmsen K, Polesie S, Gillstedt M, Osmancevic A. Impact of Etanercept on Vitamin D Status and Vitamin D-binding Protein in Bio-naïve Patients with Psoriasis. Acta dermato-venereologica. 2021;101(11).

14. Trakaki A, Wolf P, Weger W, Eichmann TO, Scharnagl H, Stadler JT, et al. Biological anti-psoriatic therapy profoundly affects high-density lipoprotein function. Biochimica et biophysica acta Molecular and cell biology of lipids. 2021;1866(7):158943.

15. Tamer F, Kucukhemek F, Gulekon A. Monocyte to high-density lipoprotein cholesterol ratio decreased in patients with psoriasis treated with ixekizumab. Revista de investigacion clinica; organo del Hospital de Enfermedades de la Nutricion. 2023;75(4):187-92.

16. Saraceno R, Rizza S, Faleri S, Federici M, Nistico SP, Copetti M, et al. High density cholesterol level as predictor of clinical response to anti-TNF-alpha therapy in psoriatic patients. Journal of biological regulators and homeostatic agents. 2013;27(3):903-8.

17. Pina T, Genre F, Lopez-Mejias R, Armesto S, Ubilla B, Mijares V, et al. Relationship of Leptin with adiposity and inflammation and Resistin with disease severity in Psoriatic patients undergoing anti-TNF-alpha therapy. Journal of the European Academy of Dermatology and Venereology. 2015;29(10):1995-2001.

18. Noe MH, Wan MT, Shin DB, Armstrong AW, Duffin KC, Chiesa Fuxench ZC, et al. Patient-reported outcomes of adalimumab, phototherapy, and placebo in the Vascular Inflammation in Psoriasis Trial: A randomized controlled study. J Am Acad Dermatol. 2019;81(4):923-30.

19. Nguyen THP, Fagerland MW, Deyab G, Hjeltnes G, Hollan I, Feinberg MW, et al. Antirheumatic therapy is not associated with changes in circulating N-terminal pro-brain natriuretic peptide levels in patients with autoimmune arthritis. PloS one. 2021;16(6 June).

20. Megna M, Fornaro L, Potestio L, Luciano MA, Nocerino M, Delfino M, et al. Efficacy and Safety of Anti-TNF Biosimilars for Psoriasis in Pediatric and Geriatric Populations: A 72-Week Real-Life Study. Psoriasis: Targets and Therapy. 2022;12:199-204.

21. Martinez-Lopez A, Blasco-Morente G, Perez-Lopez I, Tercedor-Sanchez J, Arias-Santiago S. Studying the effect of systemic and biological drugs on intima-media thickness in patients suffering from moderate and severe psoriasis. Journal of the European Academy of Dermatology and Venereology : JEADV. 2018;32(9):1492-8.

22. Maneiro JR, Souto A, Gomez-Reino JJ. Impact of treatment with TNF antagonists on total cholesterol in patients with ankylosing spondylitis and psoriatic arthritis. Clinical rheumatology. 2017;36(5):1167-72.

23. Linde A, Gerdts E, Tveit KS, Kringeland E, Midtbø H. Subclinical cardiac organ damage in patients with moderate to severe psoriasis. Journal of clinical medicine. 2021;10(11).

24. Iervolino S, Di Minno MN, Peluso R, Lofrano M, Russolillo A, Di Minno G, et al. Predictors of early minimal disease activity in patients with psoriatic arthritis treated with tumor necrosis factor-α blockers. The Journal of rheumatology. 2012;39(3):568-73.

25. Hokstad I, Greco D, Deyab G, Fagerland MW, Agewall S, Hjeltnes G, et al. Effects of Antirheumatic Treatment on Cell Cholesterol Efflux and Loading Capacity of Serum Lipoproteins in Spondylarthropathies. Journal of clinical medicine. 2022;11(24).

26. Hoffmann JH, Knoop C, Enk AH, Hadaschik EN. Routine Laboratory Parameter Dynamics and Laboratory Adverse Events in Psoriasis Patients on Long-term Treatment with Adalimumab, Etanercept, and Ustekinumab. Acta dermato-venereologica. 2017;97(6):705-10.

27. Hassan S, Milman U, Feld J, Eder L, Lavi I, Cohen S, et al. Effects of anti-TNF-α treatment on lipid profile in rheumatic diseases: An analytical cohort study. Arthritis Research and Therapy. 2016;18(1).

28. Gordon KB, Langley RG, Leonardi C, Toth D, Menter MA, Kang S, et al. Clinical response to adalimumab treatment in patients with moderate to severe psoriasis: double-blind, randomized controlled trial and open-label extension study. J Am Acad Dermatol. 2006;55(4):598-606.

29. Gkalpakiotis S, Arenbergerova M, Gkalpakioti P, Potockova J, Arenberger P, Kraml P. Long-term impact of adalimumab therapy on biomarkers of systemic inflammation in psoriasis: Results of a 2 year study. Dermatol Ther. 2020;33(6):e14110.

30. Gkalpakiotis S, Arenbergerova M, Gkalpakioti P, Potockova J, Arenberger P, Kraml P. Impact of adalimumab treatment on cardiovascular risk biomarkers in psoriasis: Results of a pilot study. The Journal of dermatology. 2017;44(4):363-9.

31. Chen W, Ding Y, Lu J, Shi Y, Gao Y, Peng C. Efficacy and survival of infliximab in psoriasis patients: A single-center experience in China. Dermatol Ther. 2020;33(6):e14227.

32. Cauza E, Cauza K, Hanusch-Enserer U, Etemad M, Dunky A, Kostner K. Intravenous anti TNF-alpha antibody therapy leads to elevated triglyceride and reduced HDL-cholesterol levels in patients with rheumatoid and psoriatic arthritis. Wien Klin Wochenschr. 2002;114(23-24):1004-7.

33. Botelho KP, Pontes MAA, Rodrigues CEM, Freitas MVC. Prevalence of Metabolic Syndrome Among Patients with Psoriasis Treated with TNF Inhibitors and the Effects of Anti-TNF Therapy on Their Lipid Profile: A Prospective Cohort Study. Metabolic syndrome and related disorders. 2020;18(3):154-60.

34. Bissonnette R, Harel F, Krueger JG, Guertin MC, Chabot-Blanchet M, Gonzalez J, et al. TNF-α Antagonist and Vascular Inflammation in Patients with Psoriasis Vulgaris: A Randomized Placebo-Controlled Study. Journal of Investigative Dermatology. 2017;137(8):1638-45.

35. Bachelez H, Van De Kerkhof PCM, Strohal R, Kubanov A, Valenzuela F, Lee JH, et al. Tofacitinib versus etanercept or placebo in moderate-to-severe chronic plaque psoriasis: A phase 3 randomised non-inferiority trial. The Lancet. 2015;386(9993):552-61.

36. Asahina A, Ohtsuki M, Etoh T, Gu Y, Okun MM, Teixeira HD, et al. Adalimumab treatment optimization for psoriasis: Results of a long-term phase 2/3 Japanese study. Journal of Dermatology. 2015;42(11):1042-52.

37. Armesto S, Coto-Segura P, Mayorga J, Illaro A, Santos-Juanes J. Efficacy of adalimumab in the treatment of moderate-to-severe psoriasis: A retrospective study of 100 patients in daily practice. Journal of Dermatological Treatment. 2015;26(1):49-53.

38. Ahlehoff O, Hansen PR, Gislason GH, Frydland M, Bryld LE, Elming H, et al. Myocardial function and effects of biologic therapy in patients with severe psoriasis: A prospective echocardiographic study. Journal of the European Academy of Dermatology and Venereology. 2016;30(5):819-23.

39. Adenubiova E, Arenberger P, Gkalpakioti P, Arenbergerova M, Jircikova J, Dolezal T, et al. Psoriasis treatment with adalimumab in clinical practice: long-term experience in a center for biological therapy in the Czech Republic. Journal of Dermatological Treatment. 2018;29(6):579-82.


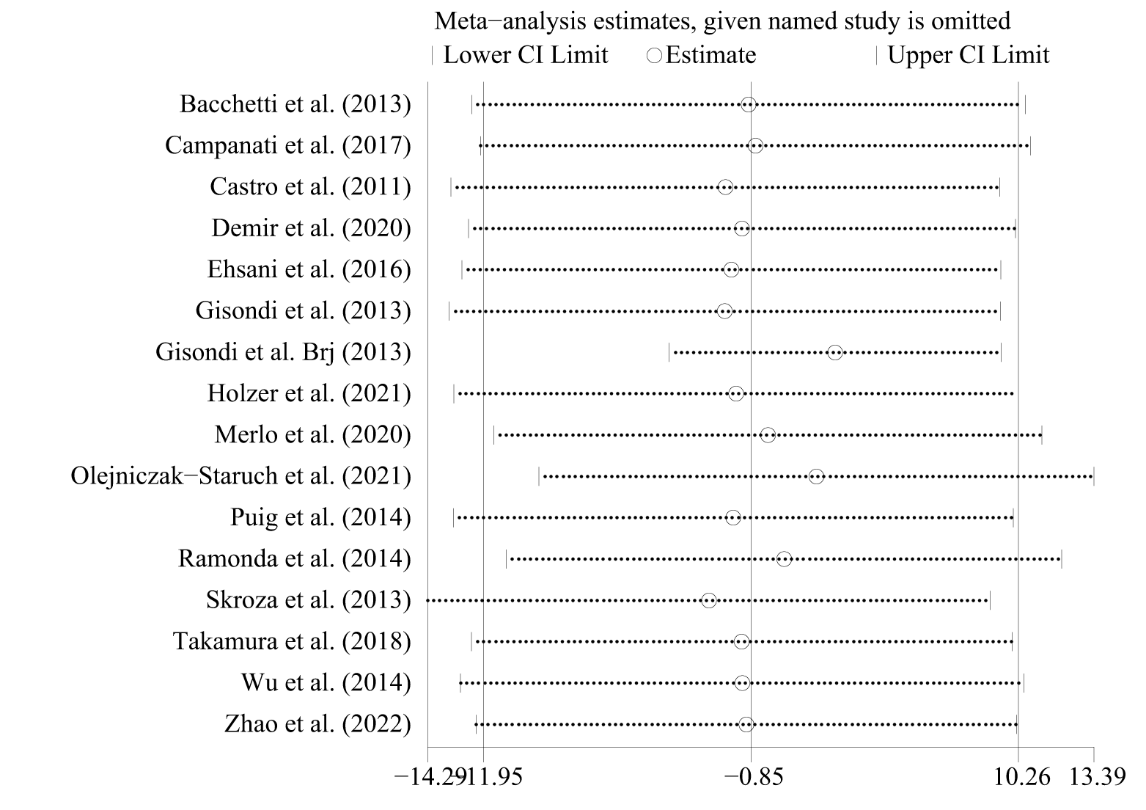


Supplemental Figure 1: Sensitivity analysis of the effects of TNF-alpha inhibitors on triglycerides.


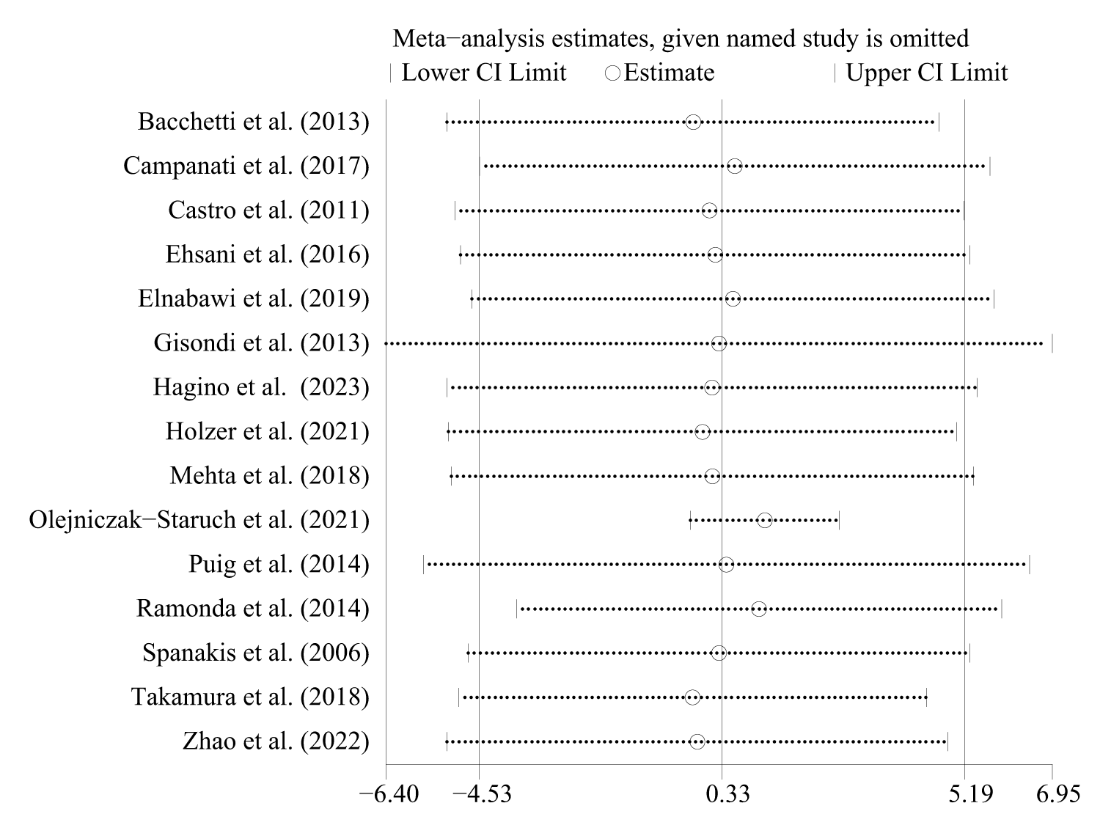


Supplemental Figure 2: Sensitivity analysis of the effects of TNF-alpha inhibitors on total cholesterol.


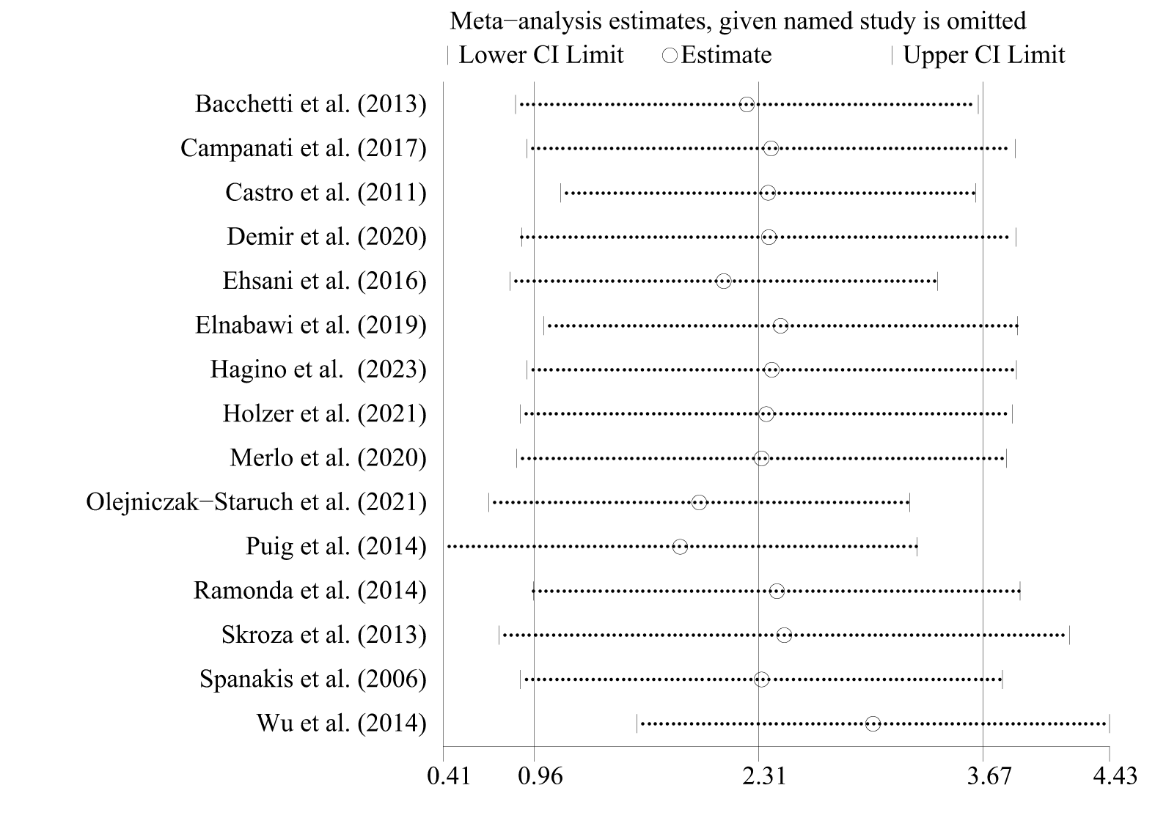


Supplemental Figure 3: Sensitivity analysis of the effects of TNF-alpha inhibitors on high-density lipoprotein.


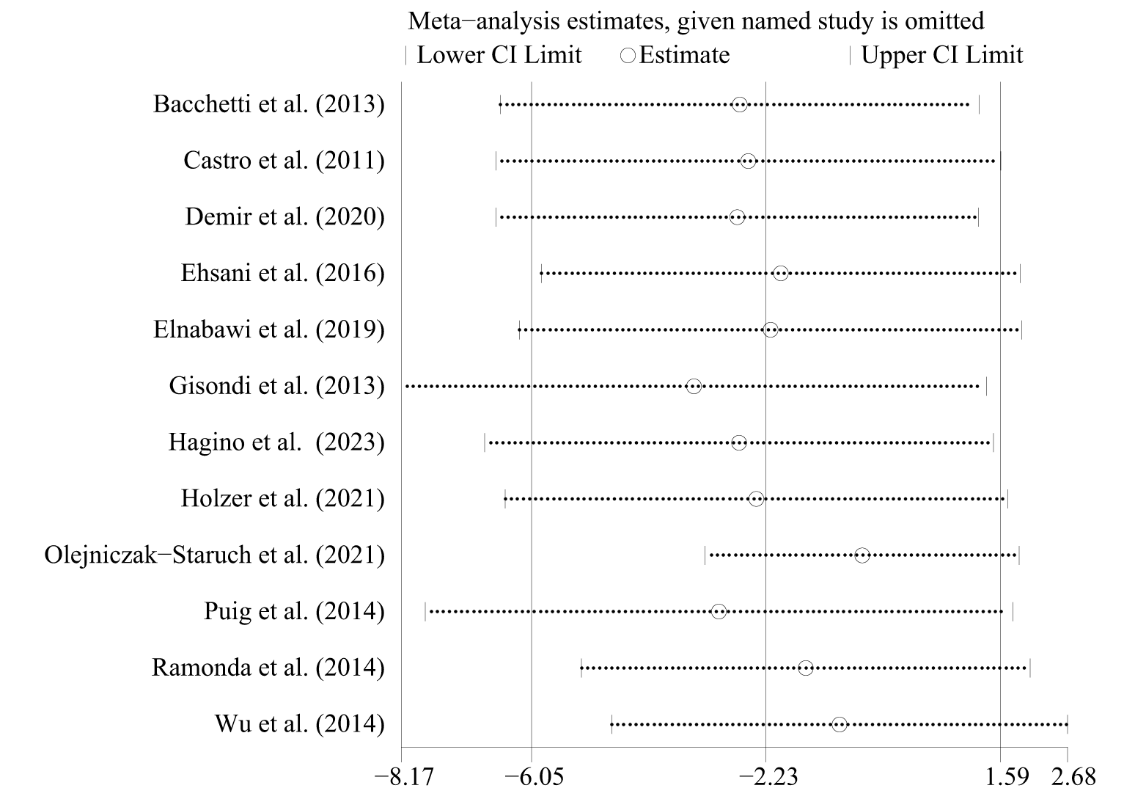


Supplemental Figure 4: Sensitivity analysis of the effects of TNF-alpha inhibitors on low-density lipoprotein.


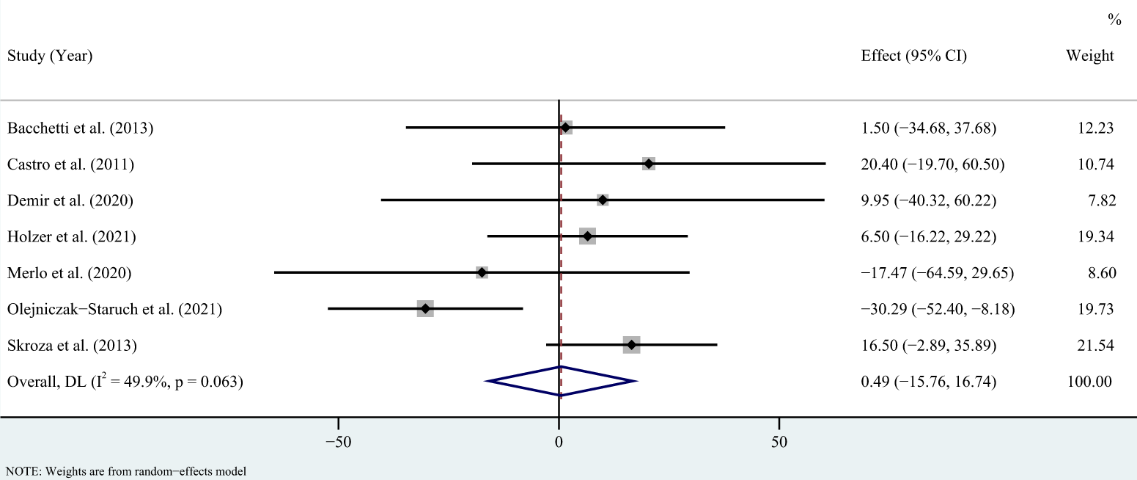


Supplemental Figure 5: Additional sensitivity analysis of the effects of TNF-alpha inhibitors on triglycerides.


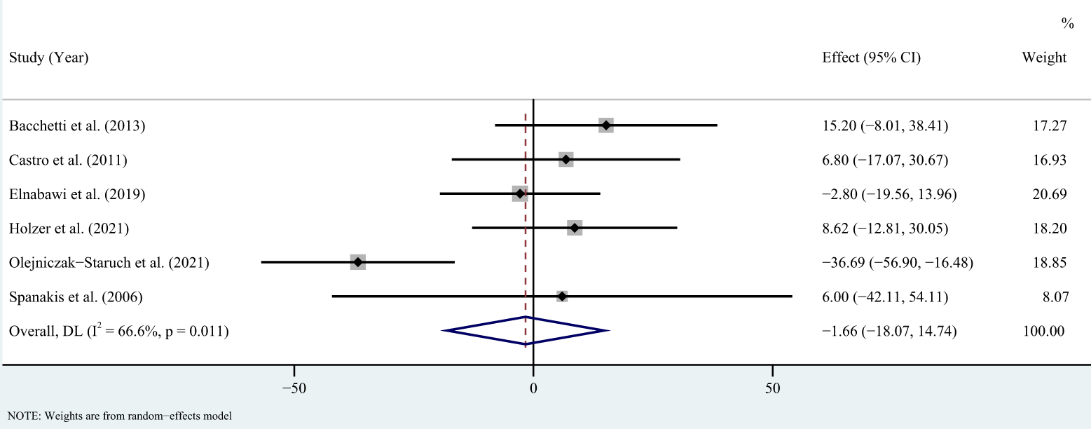


Supplemental Figure 6: Additional sensitivity analysis of the effects of TNF-alpha inhibitors on total cholesterol.


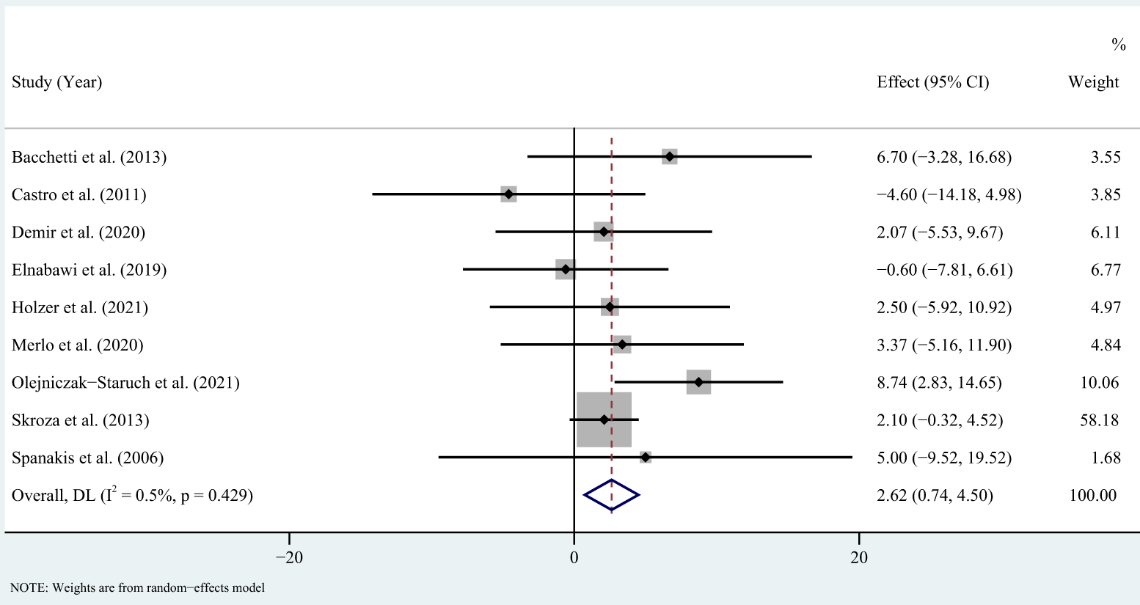


Supplemental Figure 7: Additional sensitivity analysis of the effects of TNF-alpha inhibitors on high-density lipoprotein.


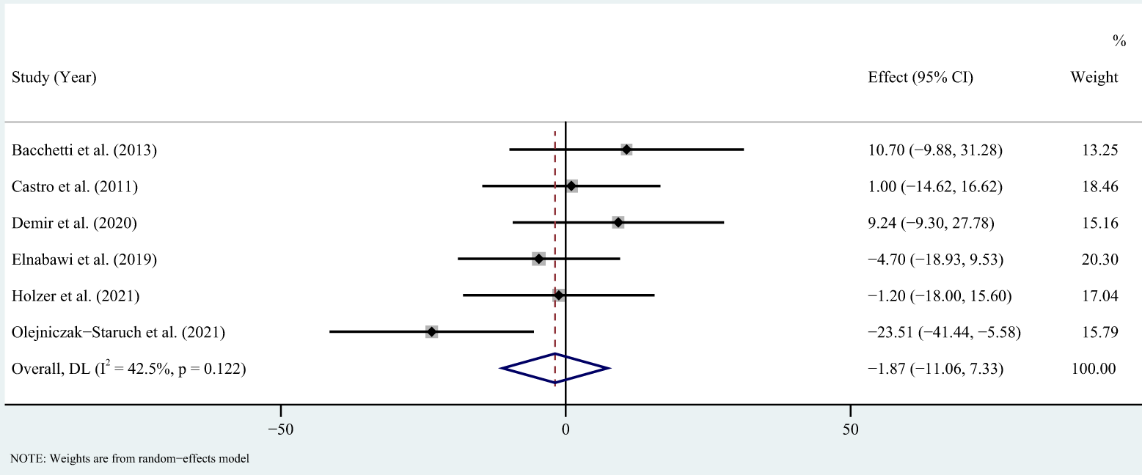


Supplemental Figure 8: Additional sensitivity analysis of the effects of TNF-alpha inhibitors on low-density lipoprotein.


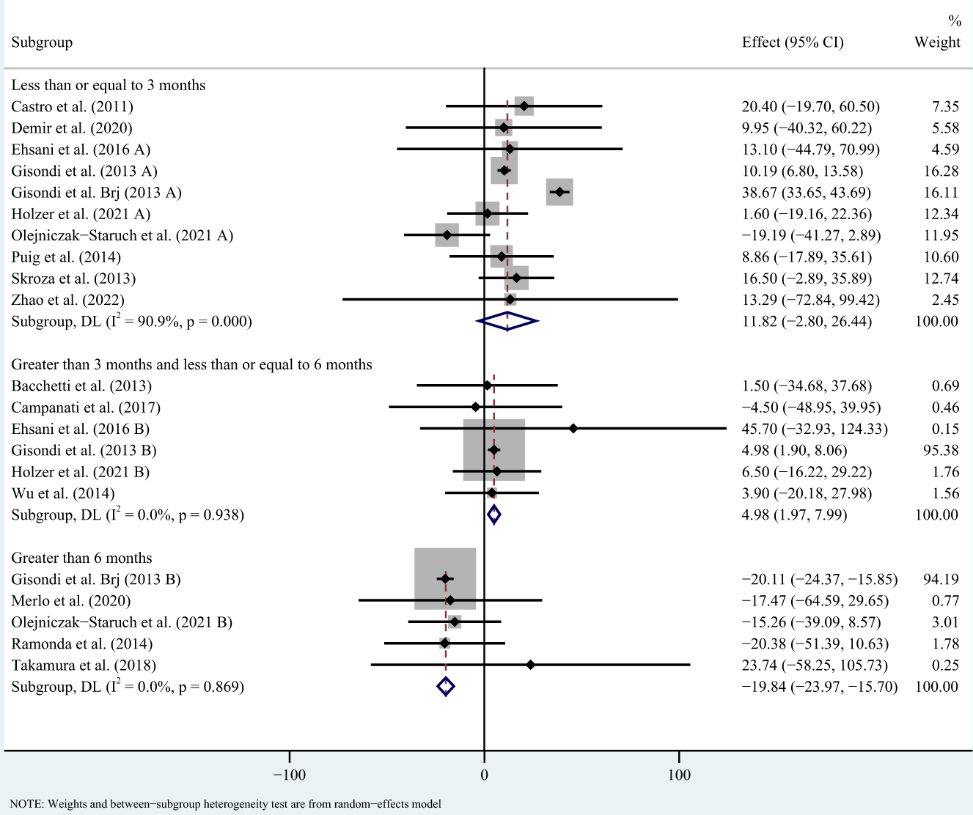


Supplemental Figure 9: Subgroup analysis according to duration of intervention (triglycerides).


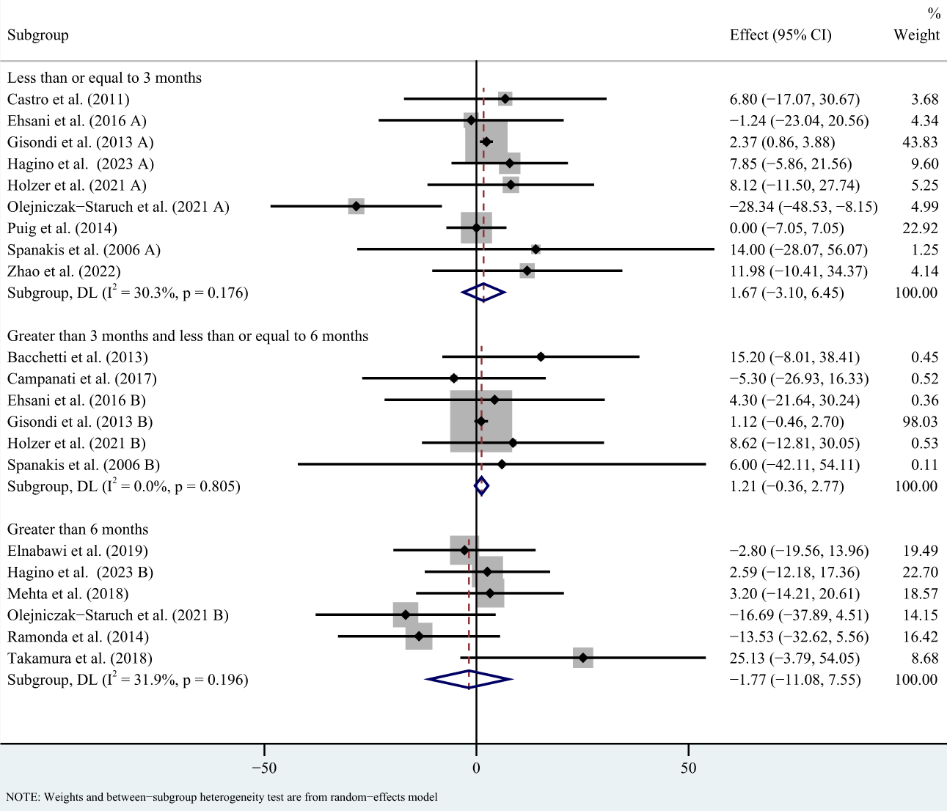


Supplemental Figure 10: Subgroup analysis according to duration of intervention (total cholesterol).


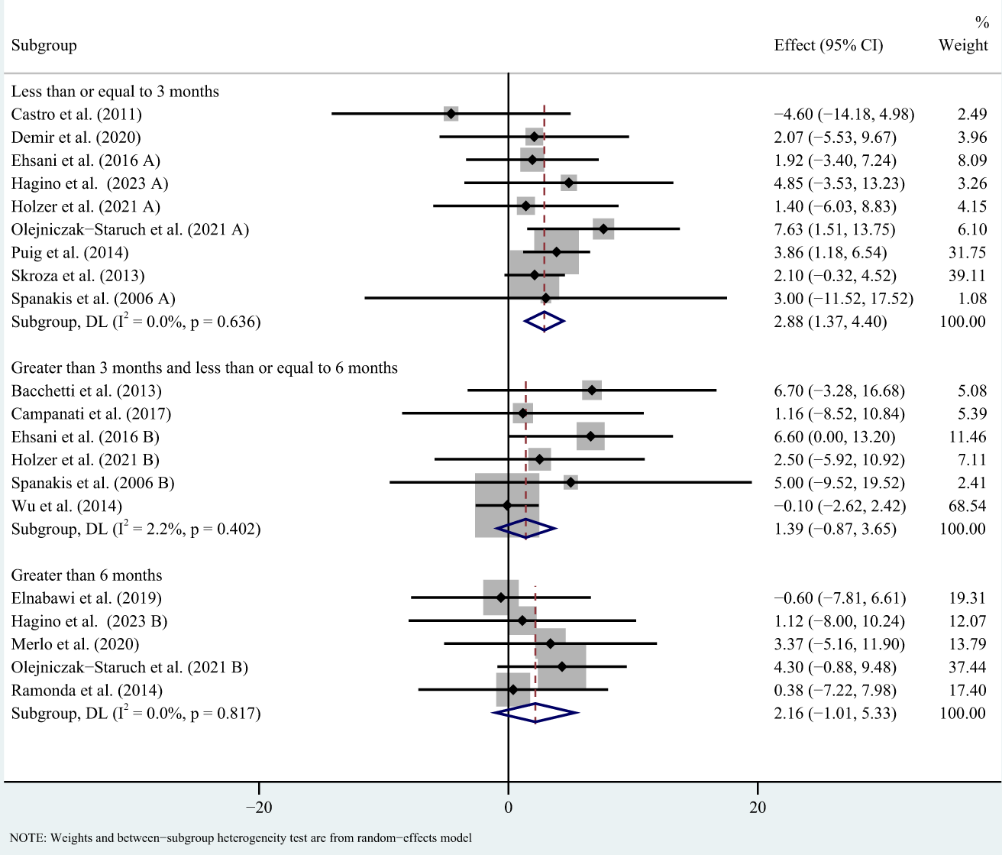


Supplemental Figure 11: Subgroup analysis according to duration of intervention (high-density lipoprotein).


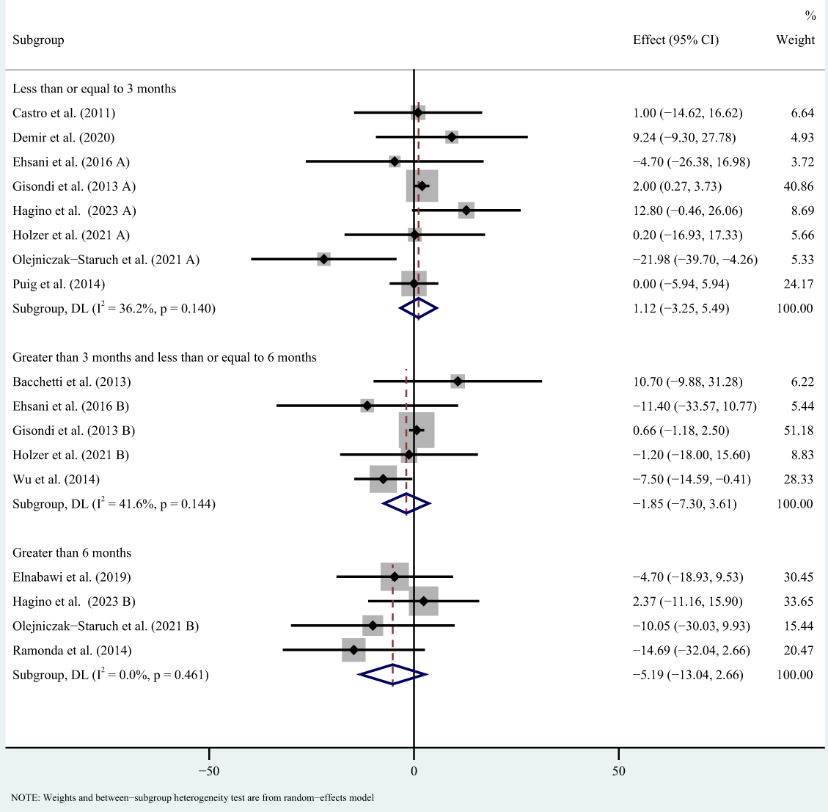


Supplemental Figure 12: Subgroup analysis according to duration of intervention (low-density lipoprotein).


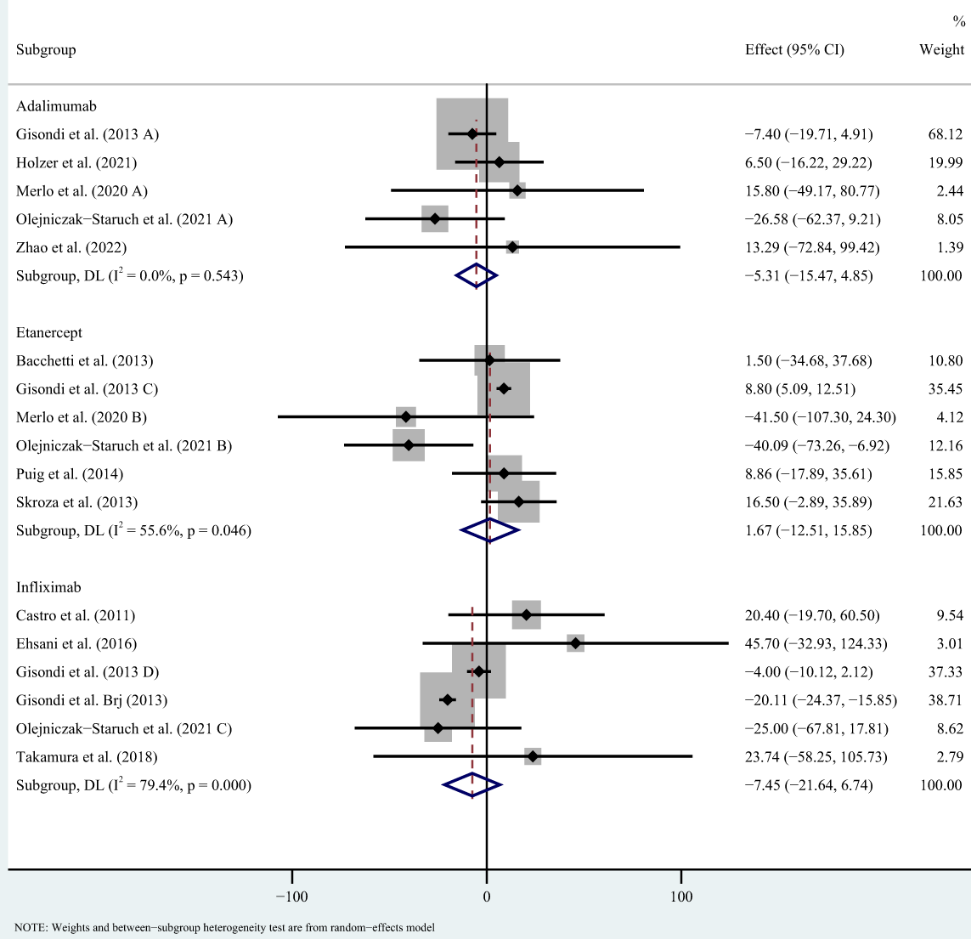


Supplemental Figure 13: Subgroup analysis according to type of TNF-alpha inhibitor (triglycerides).


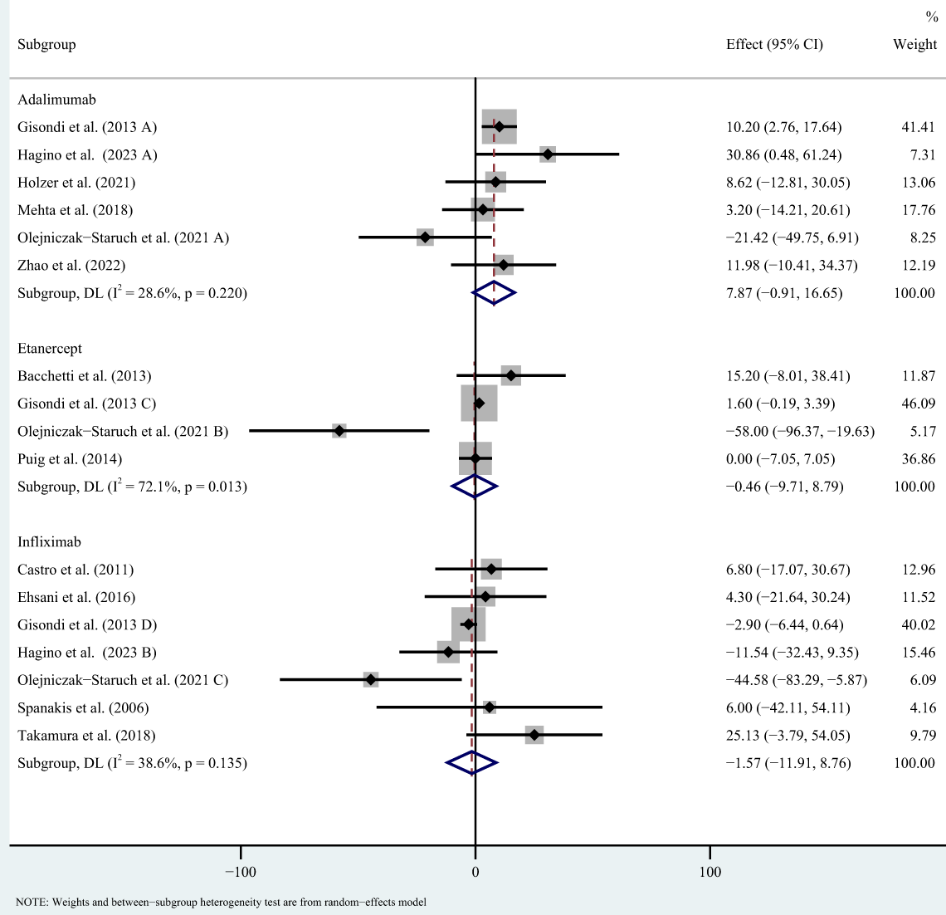


Supplemental Figure 14: Subgroup analysis according to type of TNF-alpha inhibitor (total cholesterol).


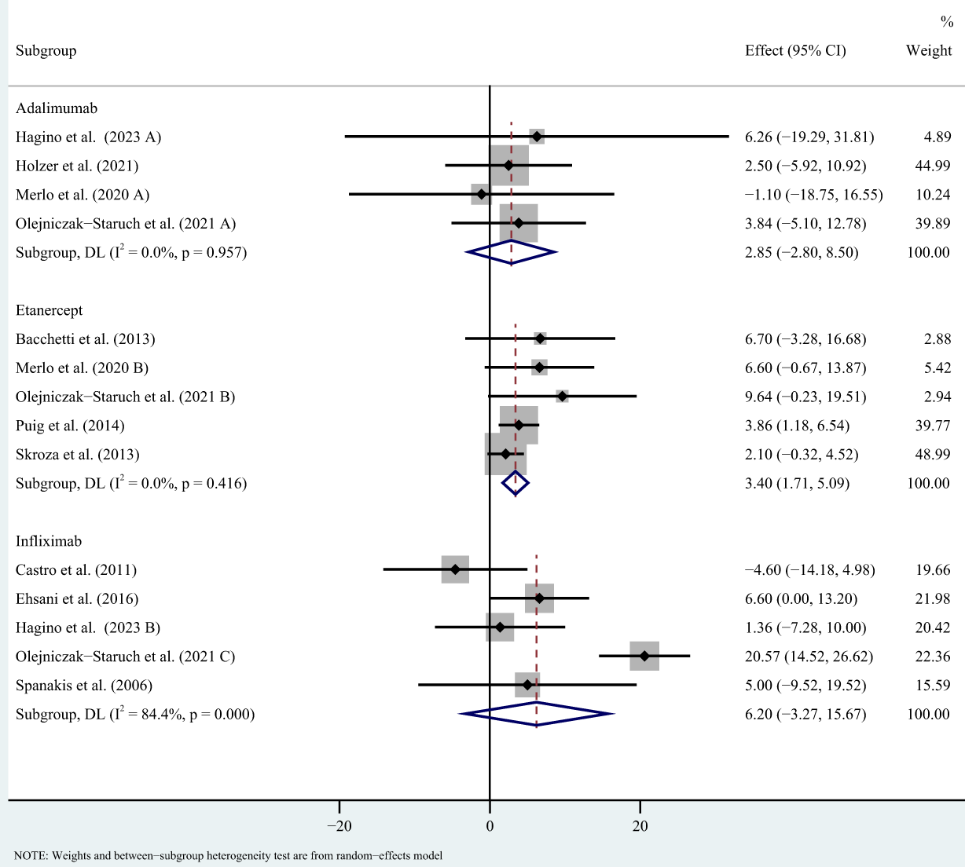


Supplemental Figure 15: Subgroup analysis according to type of TNF-alpha inhibitor (high-density lipoprotein).


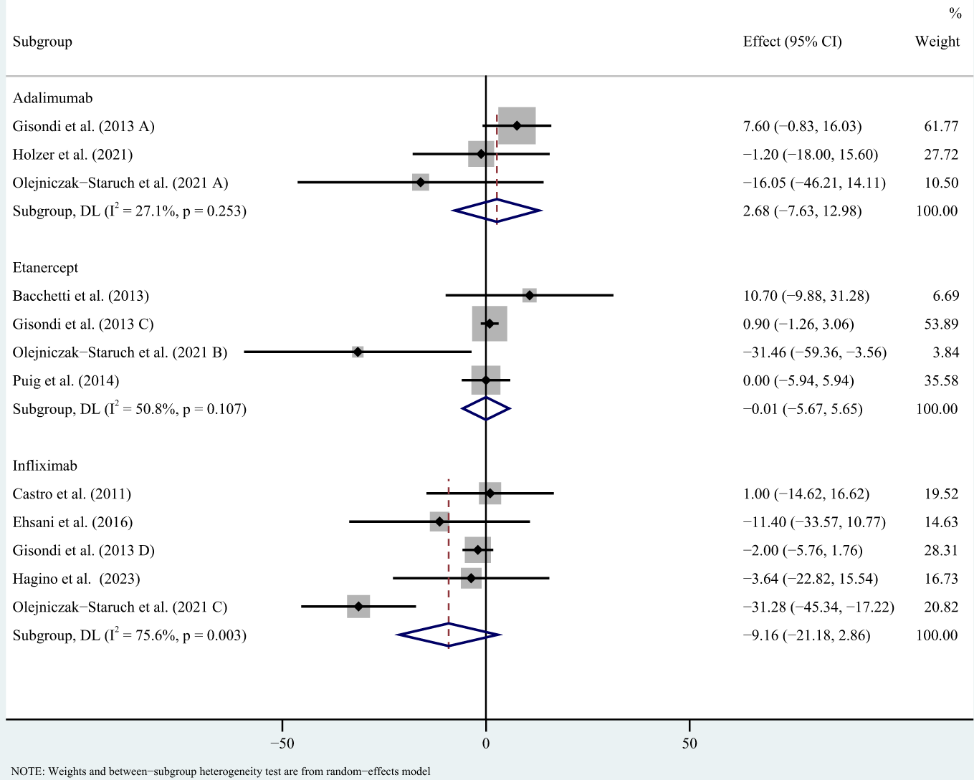


Supplemental Figure 16: Subgroup analysis according to type of TNF-alpha inhibitor (low-density lipoprotein).


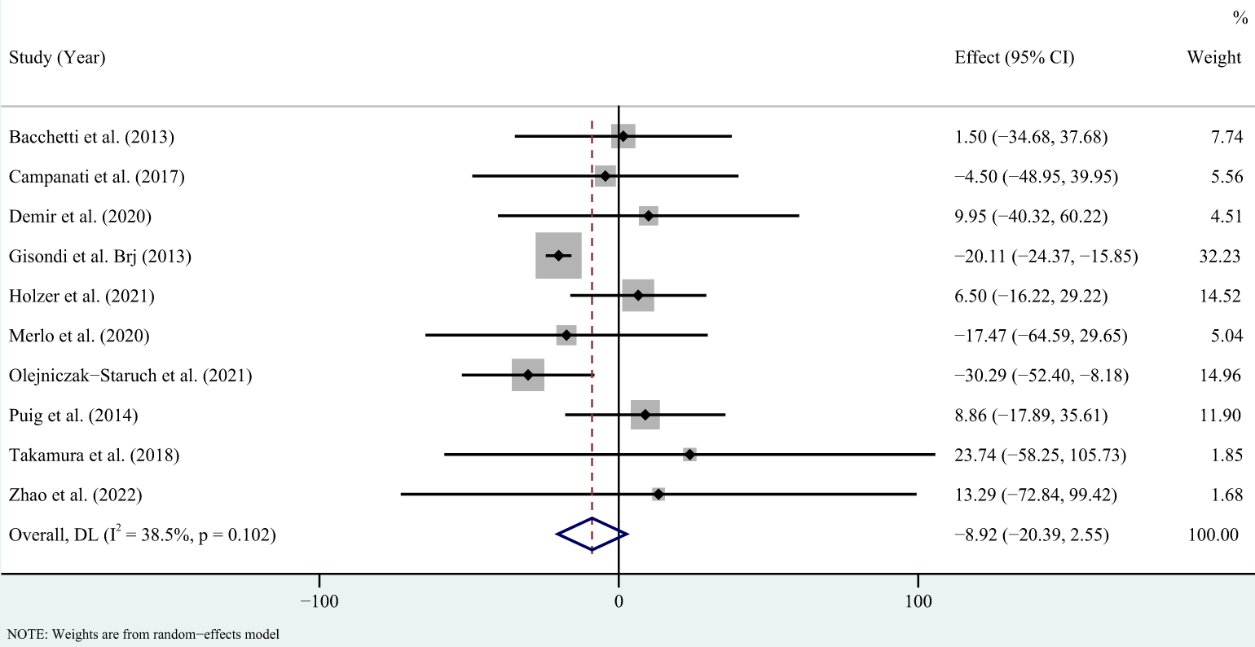


Supplemental Figure 17: Additional sensitivity analysis according to the PASI scores (triglycerides).


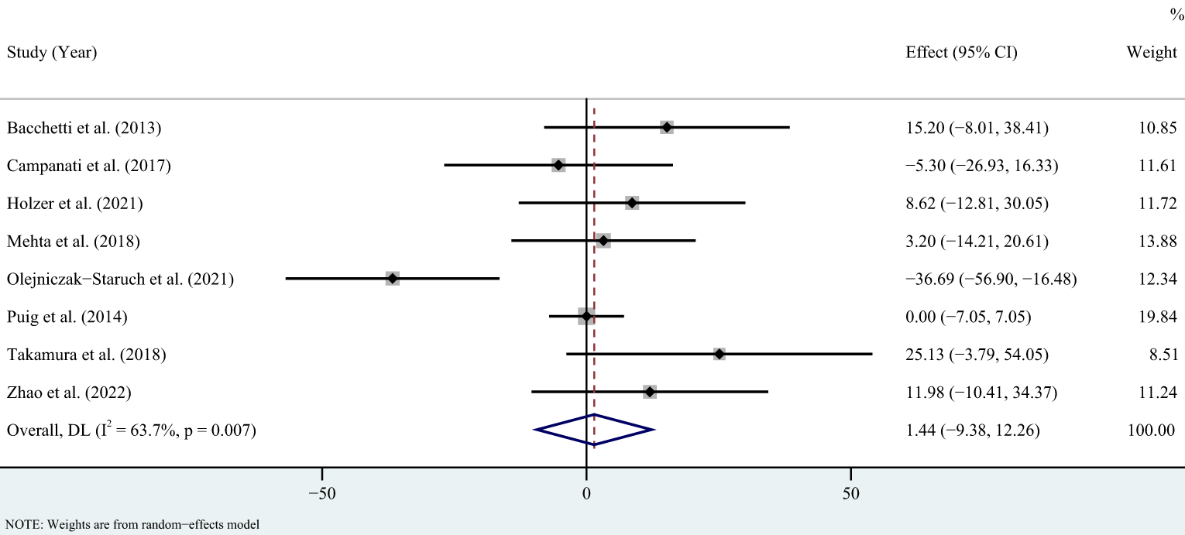


Supplemental Figure 18: Additional sensitivity analysis according to the PASI scores (total cholesterol).


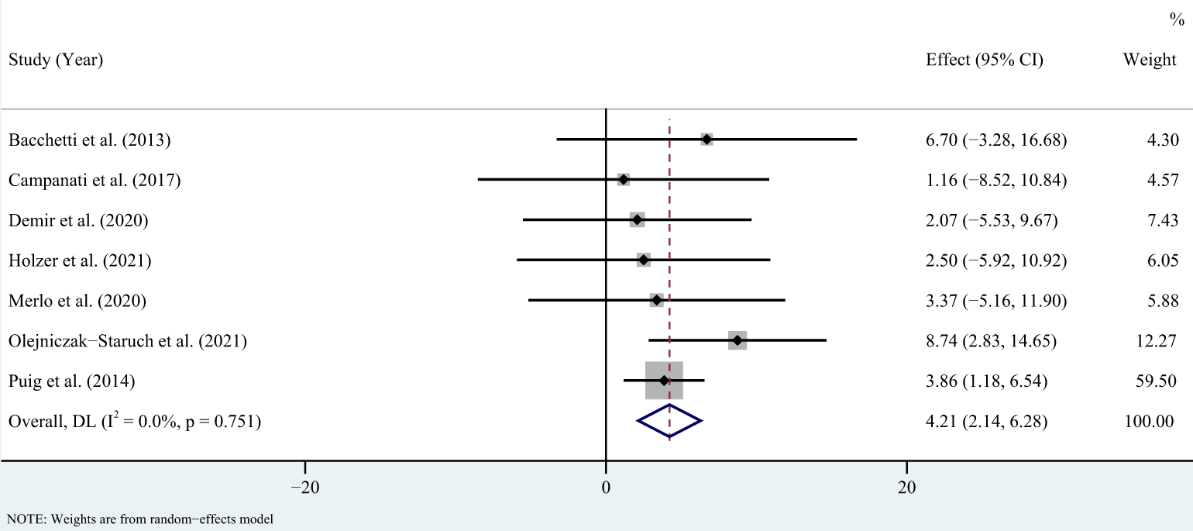


Supplemental Figure 19: Additional sensitivity analysis according to the PASI scores (high-density lipoprotein).


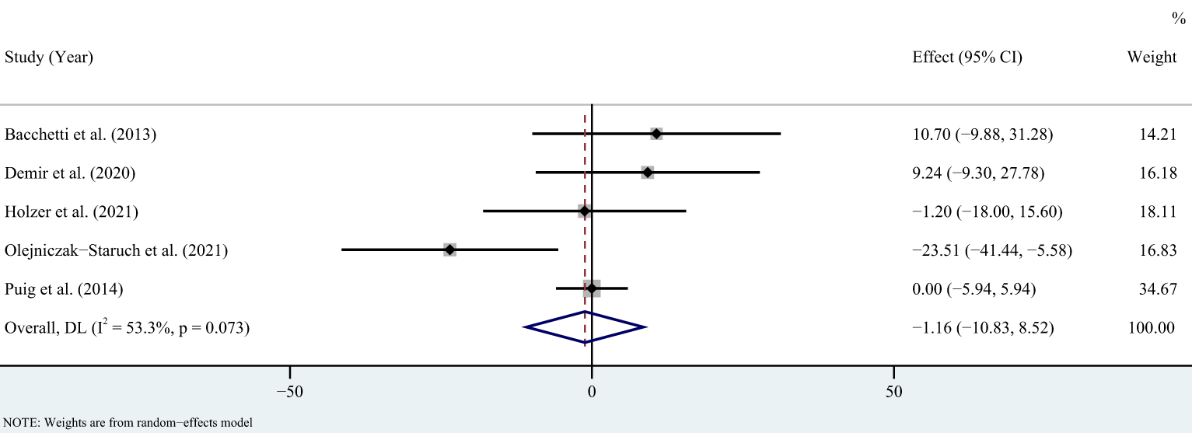


Supplemental Figure 20: Additional sensitivity analysis according to the PASI scores (low-density lipoprotein).


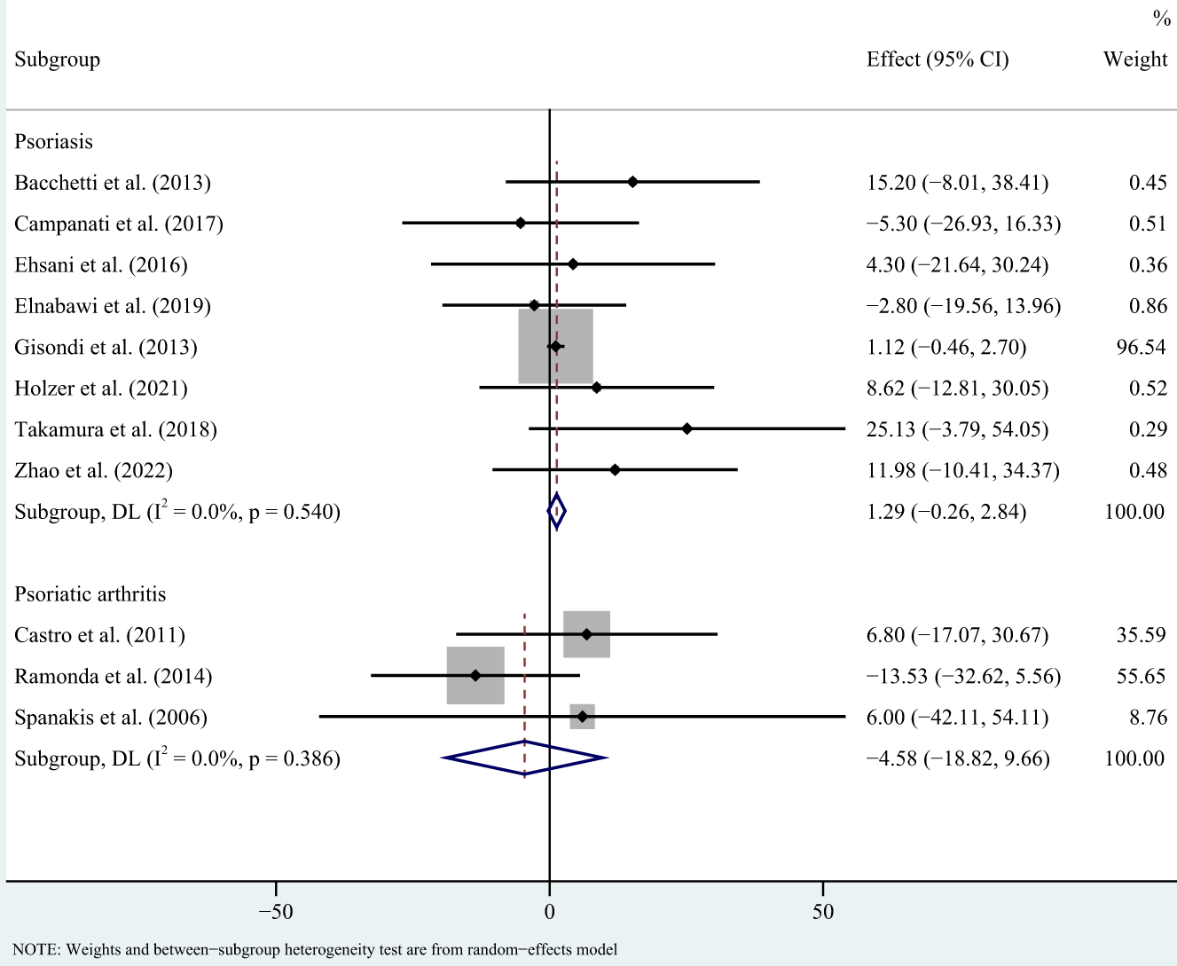


Supplemental Figure 21: Additional sensitivity analysis according to the psoriasis type (total cholesterol).


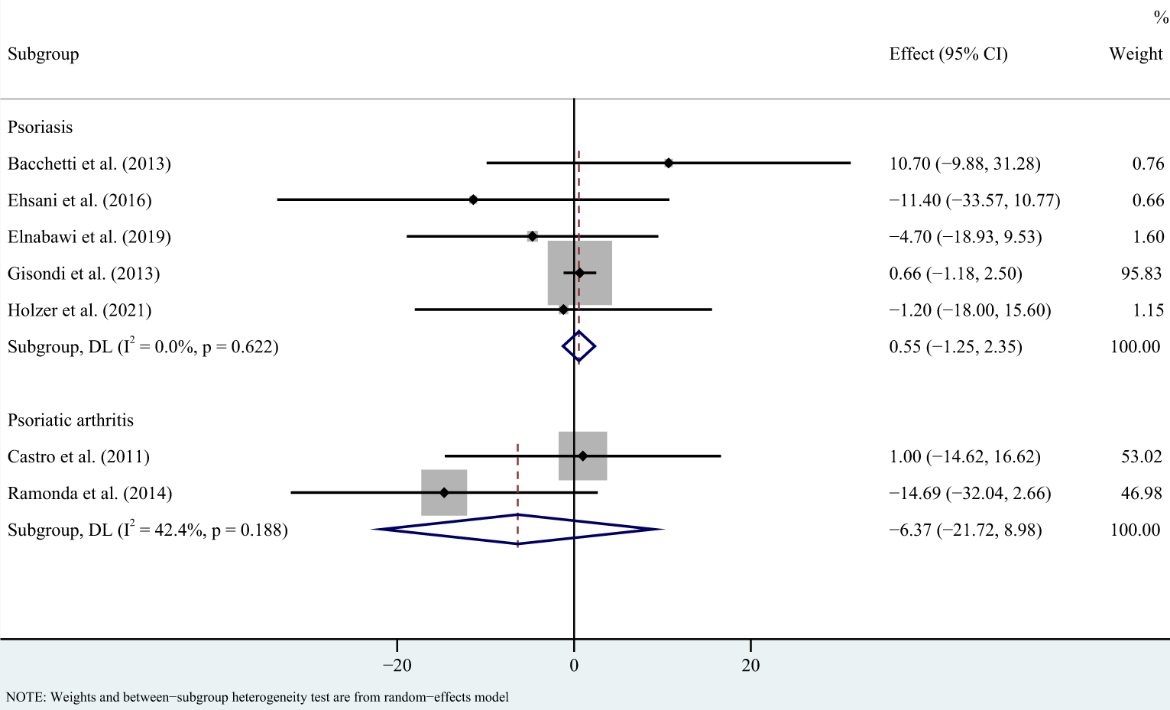


Supplemental Figure 22: Additional sensitivity analysis according to the psoriasis type (low-density lipoprotein).


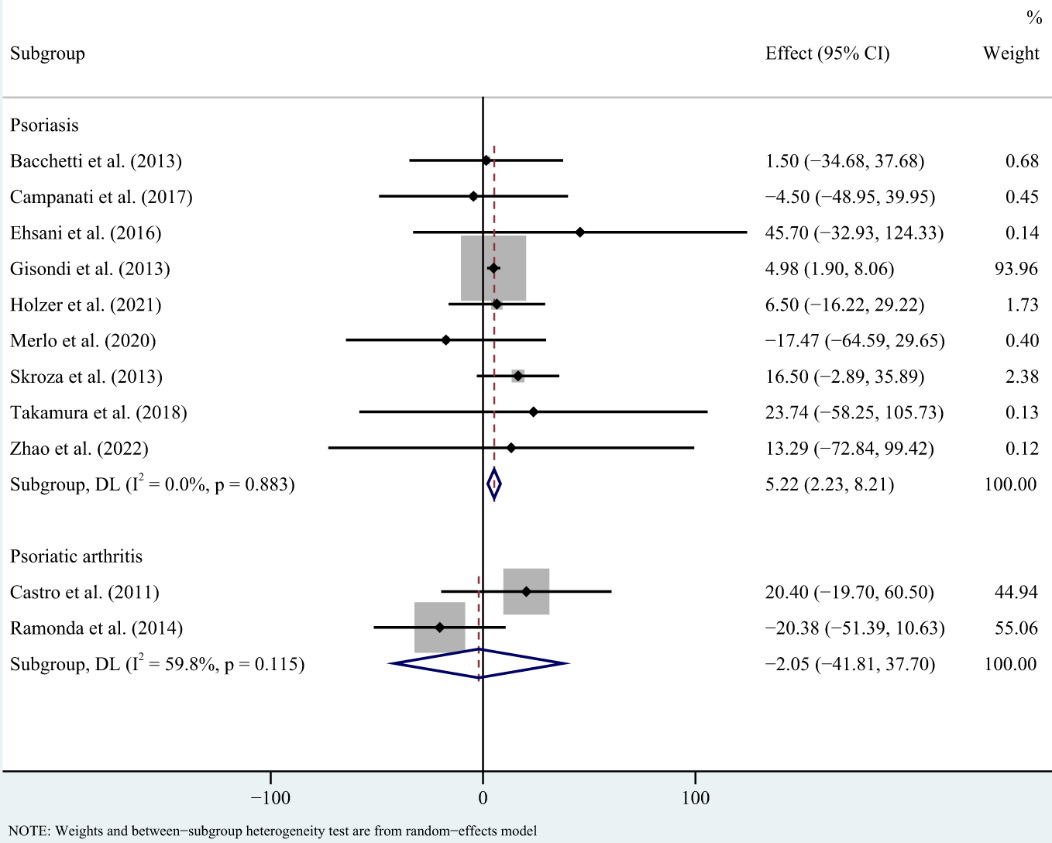


Supplemental Figure 23: Additional sensitivity analysis according to the psoriasis type (triglycerides).


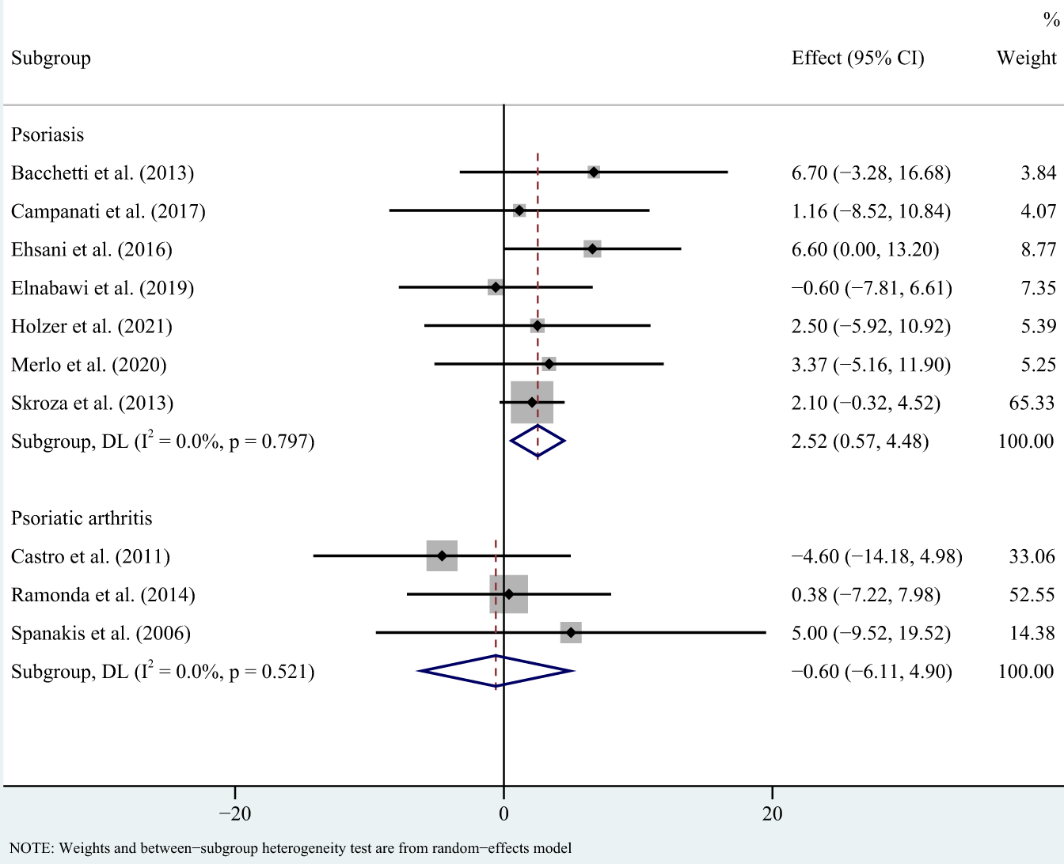


Supplemental Figure 24: Additional sensitivity analysis according to the psoriasis type (high-density lipoprotein).


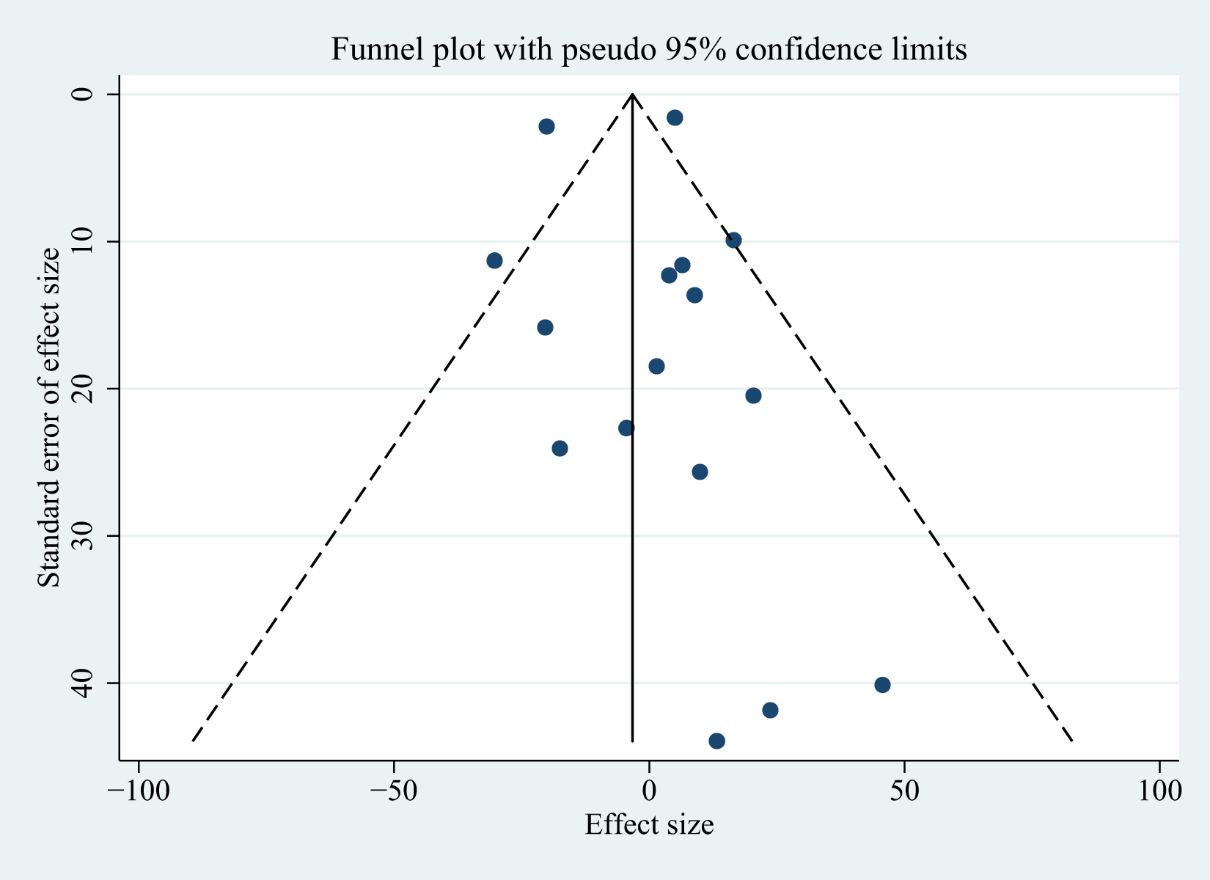


Supplemental Figure 25: The funnel plots of the effects of TNF-alpha inhibitors on triglycerides.


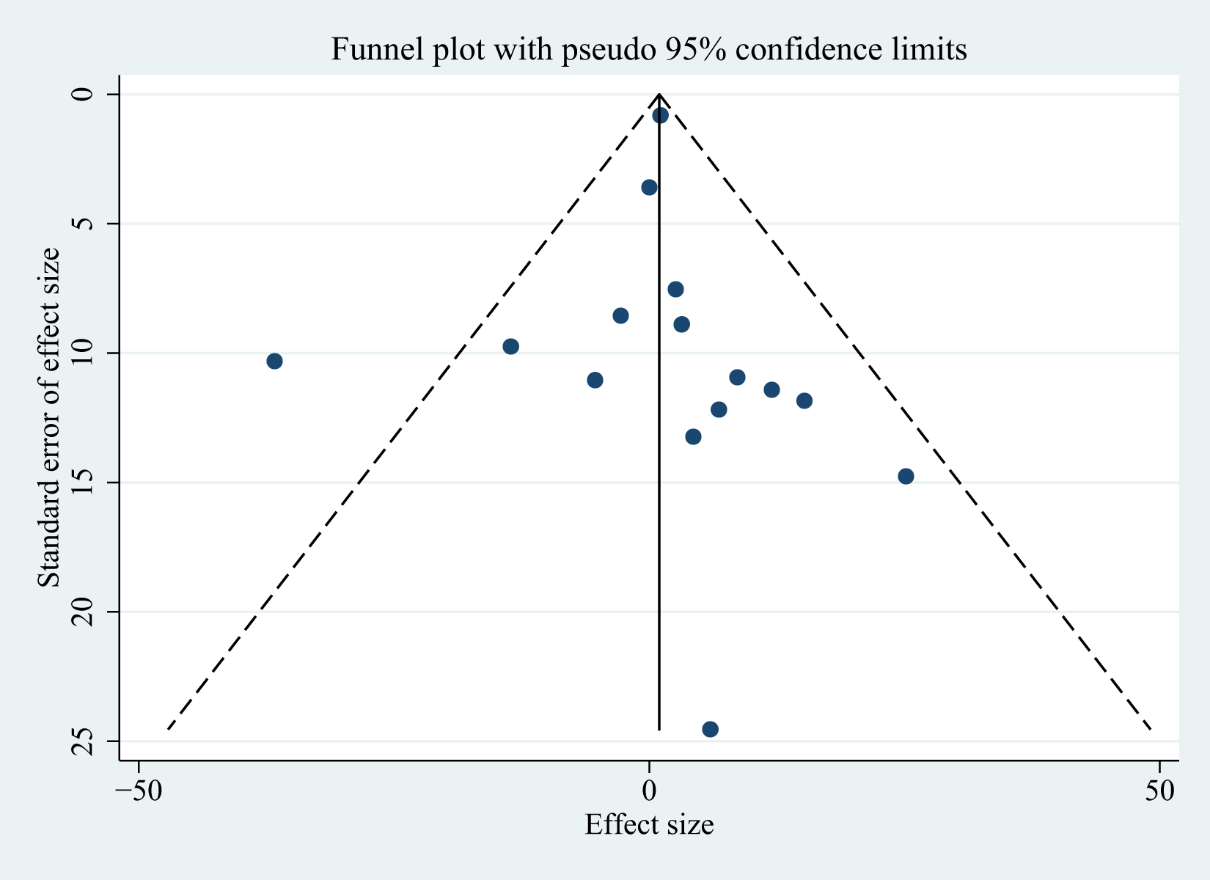


Supplemental Figure 26: The funnel plots of the effects of TNF-alpha inhibitors on total cholesterol.


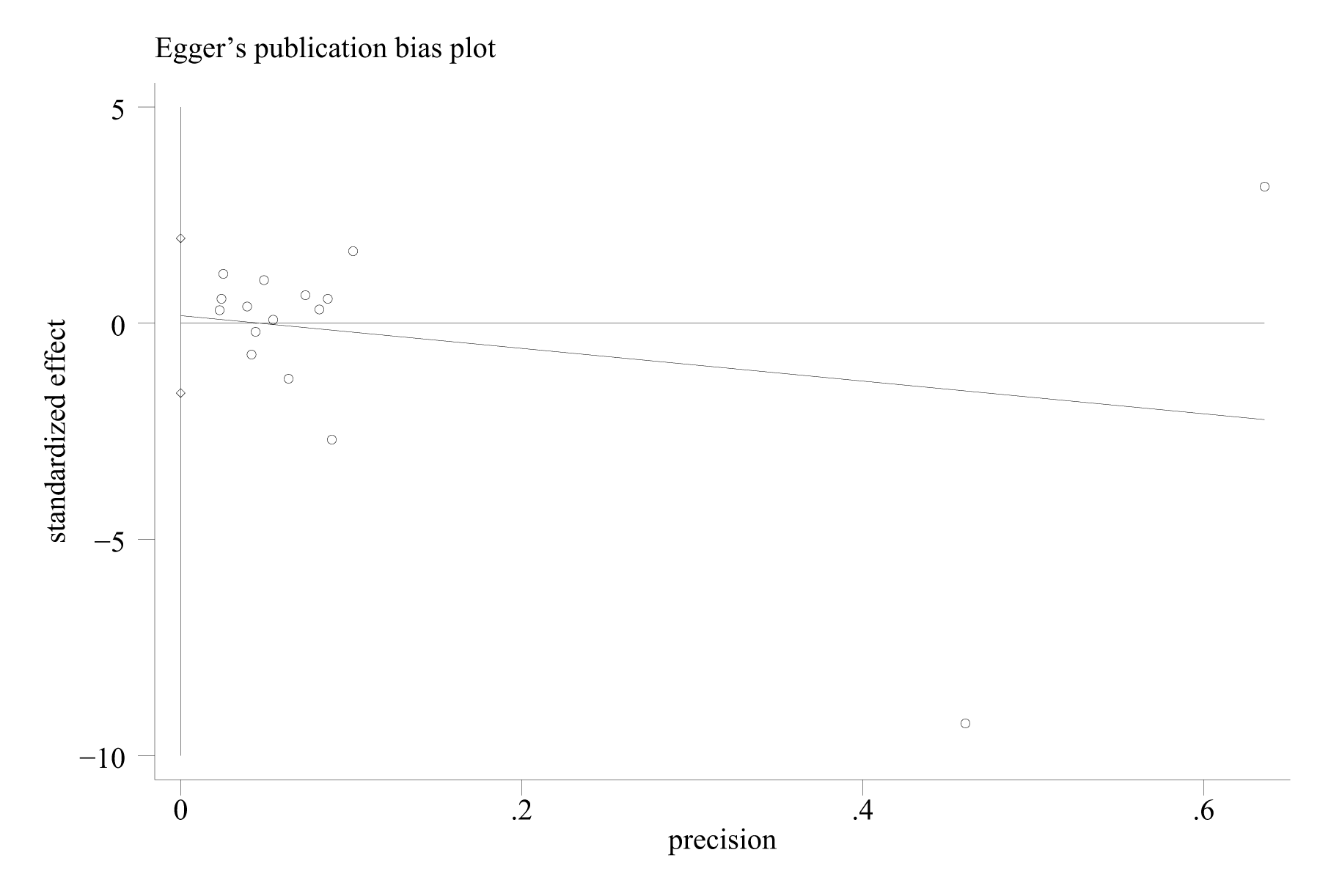


Supplemental Figure 27: The Egger’s test of the effects of TNF-alpha inhibitors on triglycerides.


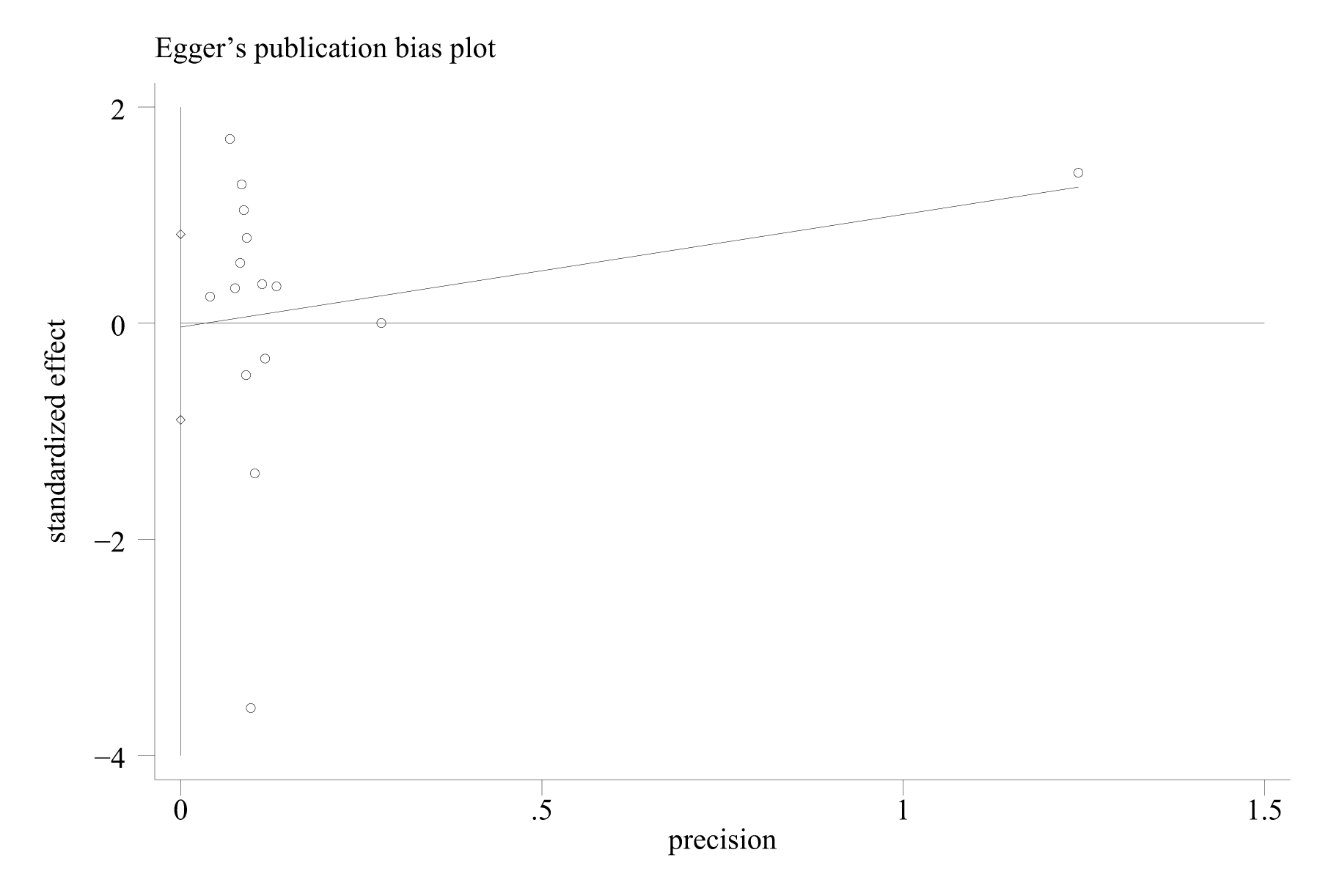


Supplemental Figure 28: The Egger’s test of the effects of TNF-alpha inhibitors on total cholesterol.


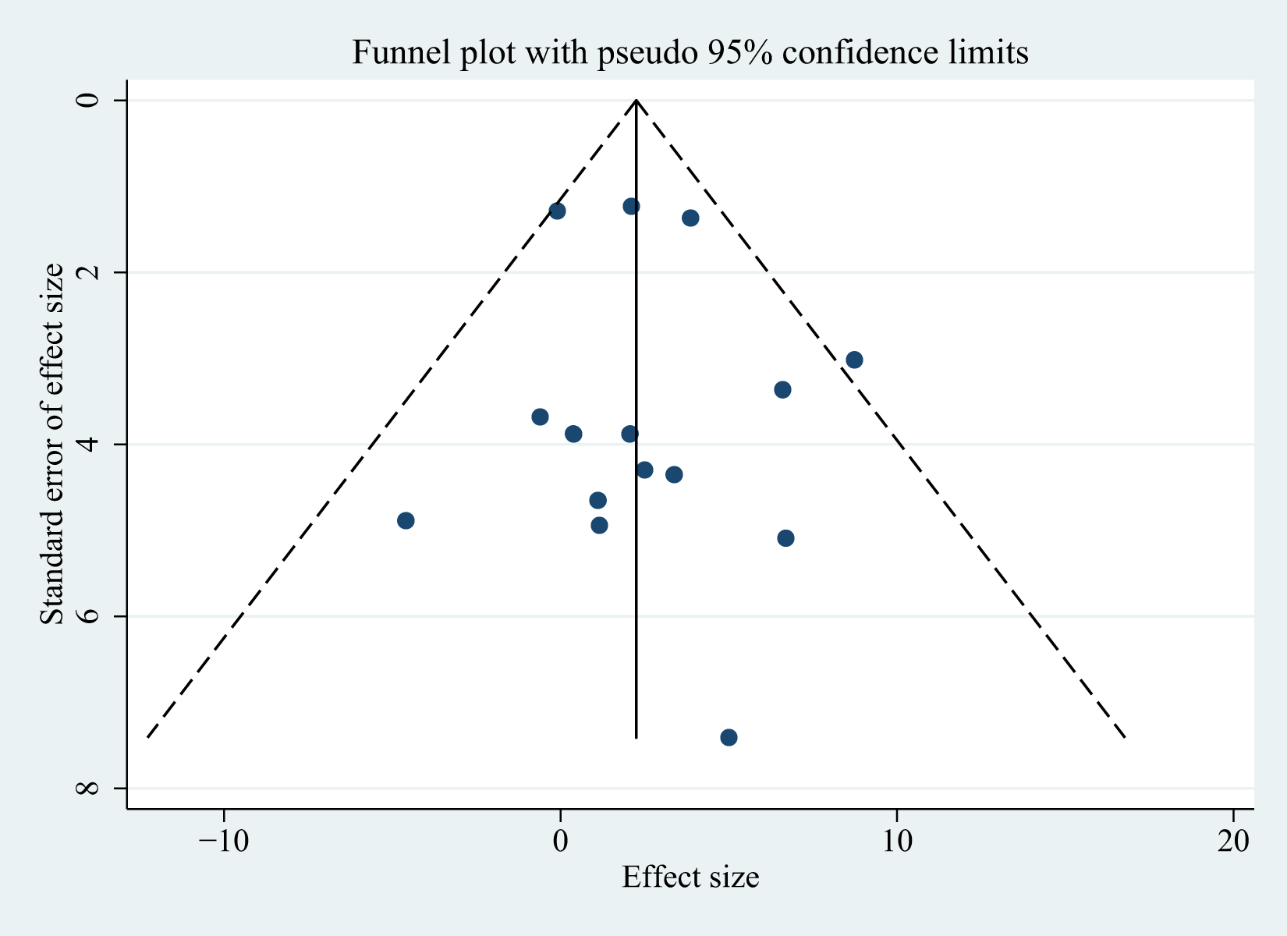


Supplemental Figure 29: The funnel plots of the effects of TNF-alpha inhibitors on high-density lipoprotein.


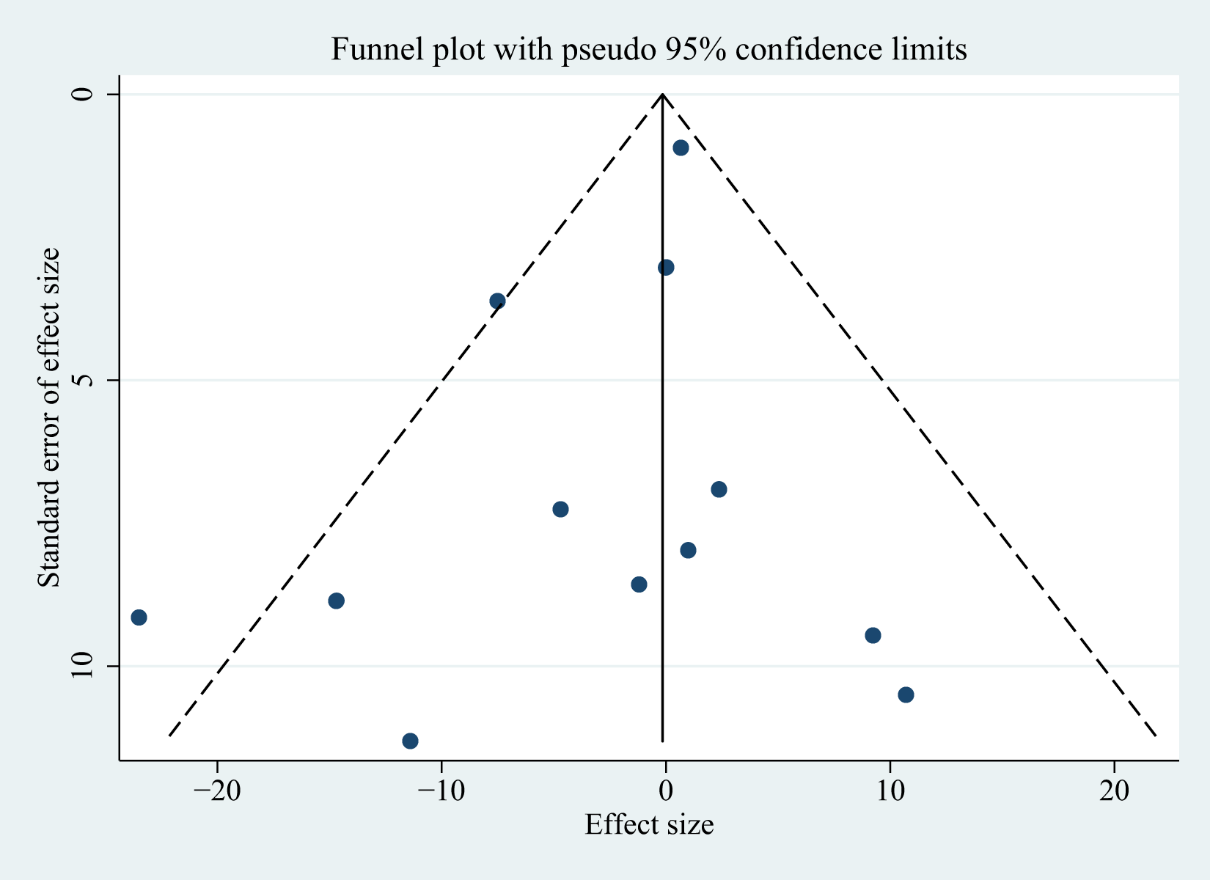


Supplemental Figure 30: The funnel plots of the effects of TNF-alpha inhibitors on low-density lipoprotein.


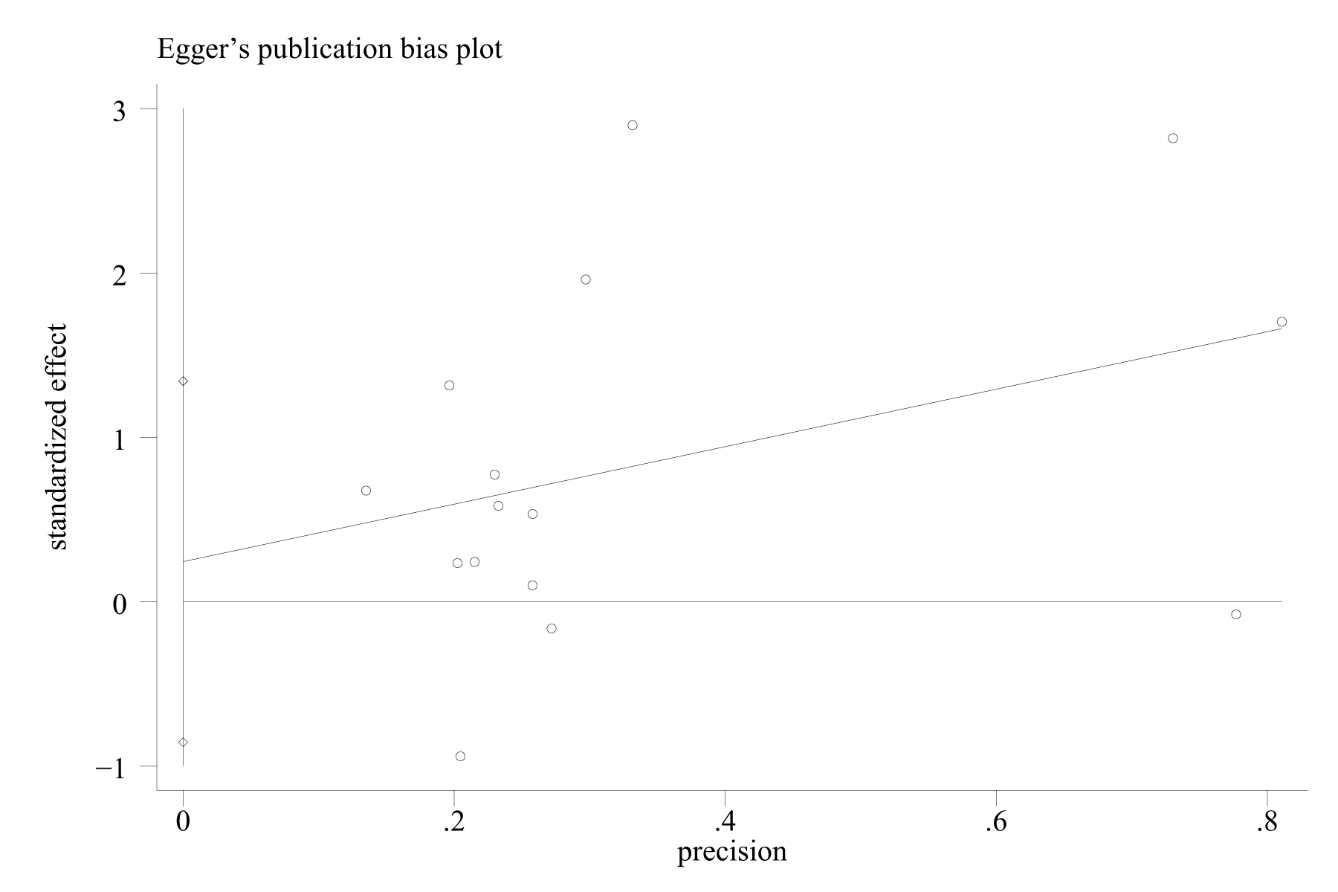


Supplemental Figure 31: The Egger’s test of the effects of TNF-alpha inhibitors on high-density lipoprotein.


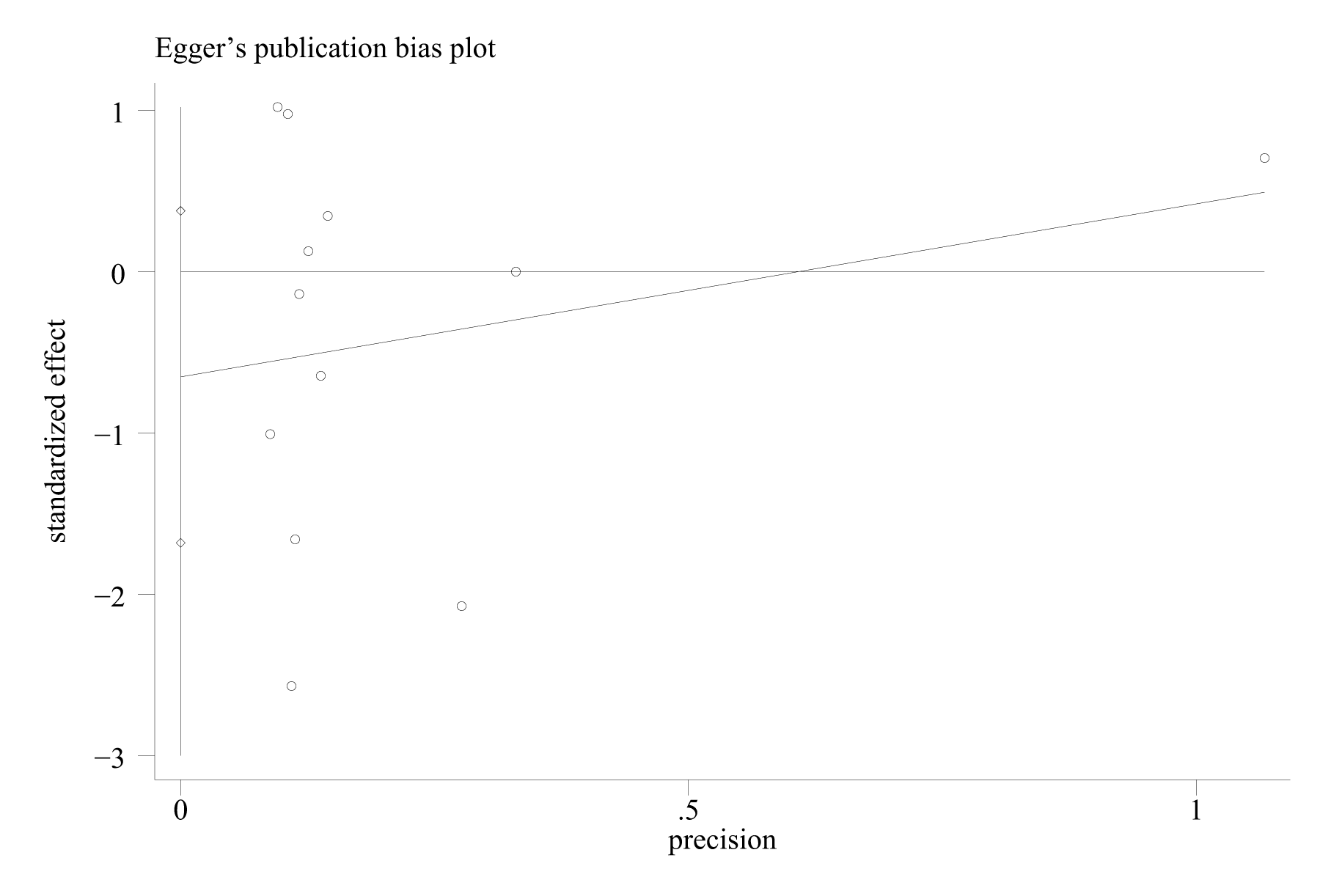


Supplemental Figure 32: The Egger’s test of the effects of TNF-alpha inhibitors on low-density lipoprotein.
